# Multivariate comparative assessment of extracts from seven *Sambucus* species reveals phytochemical diversity and biological potential

**DOI:** 10.1038/s41598-026-50152-7

**Published:** 2026-04-28

**Authors:** Aleksandra Owczarek-Januszkiewicz, Anna Magiera, Sebastian Granica, Gabriela Cieślak, Magdalena Życka, Izabela Rychlińska, Monika Anna Olszewska

**Affiliations:** 1 Department of Pharmacognosy, Faculty of Pharmacy, Medical University of Lodz, Muszyńskiego 1, 90-151 Lodz, Poland; 2 Department of Pharmaceutical Biology, Faculty of Pharmacy, Warsaw Medical University, Banacha 1, 02-097 Warsaw, Poland

**Keywords:** Interspecific variation, Rutin, Isoquercitrin, Antioxidant, Anti-inflammatory, Neutrophils, Biochemistry, Biological techniques, Biotechnology, Drug discovery, Plant sciences

## Abstract

*Sambucus nigra* is pharmaceutically important taxon, while the potential of other *Sambucus* species remains insufficiently explored. This study provides a comparative assessment of flower and leaf extracts from seven species (*S. nigra*, *S. canadensis*, *S. caerulea*, *S. racemosa*, *S. williamsii*, *S. sibirica* and *S. kamtschatica*) to identify alternative sources of valuable raw materials. UHPLC-PDA-ESI-MS/MS profiling identified 38 constituents, primarily caffeoylquinic acid derivatives and flavonol glycosides. Quantitative analysis revealed substantial interspecific variation, with quantified phenolic content accounting for 106–651 mg/g of dry extracts. Principal Component Analysis and Hierarchical Cluster Analysis identified species-specific phenolic patterns, separating rutin-dominant species from those enriched in flavonol glucosides. The extracts displayed strong antioxidant activity against physiologically important reactive oxygen species (ROS), showing OH˙ and HClO-scavenging capacity comparable to ascorbic acid. In *ex-vivo*-stimulated human neutrophils, all extracts significantly inhibited ROS release and elastase-2 secretion in a dose-dependent manner. *Z*-score normalization and Area Under the Curve metrics were employed to obtain an integrated bioactivity evaluation. Overall, the results demonstrated considerable phytochemical diversity within the genus and identified *S. williamsii* and *S. sibirica* as particularly promising for broader applications, representing viable alternatives to *S. nigra* as a source of phenolic-rich extracts with antioxidant and anti-inflammatory potential.

**Supplementary Information:**

The online version contains supplementary material available at 10.1038/s41598-026-50152-7.

## Introduction

*Sambucus nigra* (European elder) is a widely distributed species native to Europe but also introduced to North America, Asia, New Zealand, and Australia^1^. It is known since prehistoric times as a medicinal plant, with different organs used in traditional remedies^2,3^.

Currently, only the flowers of *S. nigra* (*Sambuci flos*) have a monograph in the European Pharmacopoeia^4^. Their therapeutic efficacy is attributed to the abundant fraction of flavonoids, with dominant rutin (quercetin 3-*O*-rutinoside)^1,5,6^ having established value as a vasoprotective, antioxidant and anti-infective agent^7^. Additionally, the flowers contain high amounts of caffeoylquinic acids^1^, known for their antioxidant and antiviral properties^8,9^. Consequently, the main medical applications of *S. nigra* flowers include immune system support and the symptomatic treatment of upper respiratory tract infections^1,5^. The flowers might also be considered a promising source for isolation of quercetin 3-*O*-rutinoside^10^, which is used, e.g., as a preservative, colorant, or UV absorber^7^.

Commercially available in some European countries are also *S. nigra* leaves^11^. Due to the content of cyanogenic glycosides and lectin-type proteins, they might exhibit purgative and irritant properties when administered orally, especially in fresh form. Thus, their current application in pharmacy is limited^3,11^. However, processing (e.g. drying, heating) reduces the cyanogenic potential, and the leaves have a history of traditional use in topical applications, e.g., in wound healing, supported by recent biological studies^12,13^. Moreover, they remain a viable source of natural products as cyanogenic glycosides and proteins can be effectively degraded or removed during extraction procedures. The phenolic profile of *S. nigra* leaves resembles that of the flowers, though levels of individual constituents tend to be lower^1,6^. Nevertheless, leaves provide higher biomass yields and can be harvested over a more extended period of the growing season^6^.

*Sambucus nigra* fruits also constitute a valuable pharmaceutical raw material. Numerous in vitro and in vivo studies have demonstrated their antiviral, antibacterial, and anti-inflammatory properties^1,14^.

Beyond the European elder, the genus *Sambucus* includes other species native to Europe, North America or Asia^14^. These taxa are closely related and share many general morphological features; however, they can be distinguished by differences in traits such as leaflet morphology, inflorescence architecture, and fruit characteristics including endocarp micromorphology described in taxonomic literature^15^. From a phytochemical perspective, they may also represent complementary sources of valuable plant materials. However, available data for most *Sambucus* species remain fragmentary. Existing studies report the presence of major phenolic constituents such as caffeoylquinic acids and flavonol glycosides (including quercetin 3-*O*-rutinoside and other quercetin derivatives) in species such as *S. sibirica*, *S. williamsii*, *S. racemosa*, or *S. canadensis*, however with some interspecific differences^16–19^. Preliminary reports also indicate that the composition may differ between plant organs, with leaves in some species containing at least comparable levels of phenolics compared to flowers^18^. Nevertheless, the lack of multivariate comparative studies covering both the phytochemical and biological potential of *Sambucus* species limits the selection of those most suitable for further research.

The main objective of this study was to evaluate selected *Sambucus* species in terms of phytochemical and biological potential and to compare them with the widely utilized *S. nigra*. Given the substantial number of publications addressing interspecific variability in elder fruits^20–24^, our investigation focused on flowers and leaves, particularly as sources of flavonoids and caffeoylquinic acids. The research included a thorough phytochemical profiling using UHPLC-PDA-ESI-MS^/^MS, HPLC-PDA and spectrophotometric analyses, alongside biological assays assessing direct scavenging of physiologically important reactive oxygen species (ROS) in vitro and down-regulation of ROS production and neutrophil elastase (ELA-2) secretion in human neutrophils ex vivo. To identify species with the greatest potential, the results were subjected to multivariate statistical analysis, including Principal Component Analysis (PCA) and Hierarchical Cluster Analysis (HCA).

## Experimental

### Plant material and extraction

Flowers and leaves of selected *Sambucus* species (Table 1) were collected and authenticated in 2020 in the Arboretum (51°49′N 19°53′E), the Forestry Experimental Station of Warsaw University of Life Sciences in Rogów (Poland) from plants in long-term cultivation. The identity of plant material was confirmed by Piotr Banaszczak, a dendrology specialist, and Head of the Arboretum. The collection was conducted with the permission of the Head of the Arboretum and in accordance with institutional guidelines. No protected or endangered plant species were involved in this study.

**Table 1:** List of the *Sambucus* species and obtained extracts used in the study.

Species^a^	Plant part	Voucher specimen	Extract symbol	Extraction yield [%]
*Sambucus caerulea* Raf. (syn. *S. nigra* subsp. *caerulea* Bolli)	Leaves	KFG/SCAE_1701_L	SCAE_L	30.40
*Sambucus canadensis* L. (syn. *S. nigra* subsp. *canadensis* Bolli)	Leaves	KFG/SCAN_1701_L	SCAN_L	34.53
*Sambucus kamtschatica* E.L.Wolf (syn. *S. racemosa* subsp. *kamtschatica* Hulten)	Flowers	KFG/SKAM_1701_F	SKAM_F	25.83
	Leaves	KFG/SKAM_1701_L	SKAM_L	32.08
*Sambucus nigra* L.	Flowers	KFG/SNIG_1701_F	SNIG_F	28.78
	Leaves	KFG/SNIG_1701_L	SNIG_L	25.21
*Sambucus racemosa* L.	Flowers	KFG/SRAC_1701_F	SRAC_F	32.57
	Leaves	KFG/SARC_1701_L	SRAC_L	18.38
*Sambucus sibirica* Nakai (syn. *S. racemosa* subsp. *sibirica* H.Hara)	Flowers	KFG/SSIB_1701_F	SSIB_F	26.09
	Leaves	KFG/SSIB_1701_L	SSIB_L	22.90
*Sambucus williamsii* Hance	Flowers	KFG/SWIL_1701_F	SWIL_F	28.63
	Leaves	KFG/SWIL_1701_L	SWIL_L	24.27

^a^Accepted species names according to Plants of the World Online^25^.

For each species and plant organ, material was collected from three to six plant growing in the same collection under comparable environmental condition and at similar phenological state. The flowering cymes were collected in May during full bloom and leaves were harvested in September after ripening of the fruits. The collected material was pooled to obtain a single composite biological sample for each taxon and organ. Voucher specimens (Table 1) were deposited in the Herbarium of the Department of Pharmacognosy, Medical University of Lodz (Poland). For two of the investigated species (*S. caerulea*, *S. canadensis*), it was not possible to collect sufficient flowering material due to weak flowering during the collection period. The obtained plant materials were air-dried at room temperature, and after drying, the individual flowers were manually separated from the cymes by gentle rubbing. Subsequently, the dried flowers and leaves were finely powdered using electric grinder and sieved (0.315 mm).

### Extract preparation

Samples of the dried materials (5 g) were extracted for to 24 h in a Soxhlet apparatus with chloroform (300 mL) to remove lipophilic interferences. The defatted residues were subsequently extracted with 70% (*v/v*) methanol (3 × 150 mL, 1 h). The obtained extracts (Table 1) were first concentrated under reduced pressure (Rotavapor R-200, Büchi, Flawil, Switzerland) to remove methanol and the majority of water and subsequently frozen and lyophilized to obtain completely dry samples. For each pooled sample, a single dry extract was prepared. The extracts were stored at 4 °C until analysis.

### Qualitative UHPLC-PDA-ESI-MS/MS analysis

Qualitative profiling was performed on a UHPLC-3000 RS system (Dionex, Dereich, Germany) equipped with a binary pump, column oven, autosampler, diode array detector and an AmaZon SL unit-resolution ion trap mass spectrometer with an ESI source (Bruker Daltonik, Bremen, Germany). Separations were carried out on a Kinetex XB-C18 column (1.7 µm, 150 mm × 2.1 mm i.d.; Phenomenex, Torrance, CA, USA) at 25 °C. The mobile phase consisted of 0.1% (*v/v*) formic acid (Solvent A) and acetonitrile (solvent B), both HPLC grade (Avantor Performance Materials, Gliwice, Poland). The gradient program was: 0–0.5 min, 6% B in A; 0.5–45 min; 6–26% B; 45–55 min, 26–95% B; 55–65 min, 95% B; 65–65.01 min, 95–6% B; 65.01–75 min 6% B. The flow rate was 0.3 mL/min. Extract solutions (10 mg/mL) were filtered through a PTFE syringe filter (13 mm, 0.2 µm, Whatman, Pittsburgh, PA, USA). UV–Vis spectra were recorded from 200 to 600 nm. The LC-eluate was introduced directly into the ESI source and analyzed in both negative and positive modes. Operating parameters were: nebulizer pressure, 40 psi; dry gas flow, 9 L/min; dry temperature, 300 °C; capillary voltage, 4.5 kV. Fragmentations data were acquired in Auto MS/MS mode (collision energy selected automatically based on the characteristics of the precursor ion) for the most abundant ions. The scan range was 200–2000 m*/z*. Compound identification was based on a combination of chromatographic behaviors and MS/MS fragmentation patterns. Whenever possible reference standards were used for comparison. Additionally, the presence of sambunigrin was assessed qualitatively by comparison of its retention and characteristic fragmentation pattern with these of an authentic reference standard analyzed under the same LC-MS conditions.

### Quantitative HPLC analysis of polyphenols

Quantitative determination of phenolic compounds was performed using a Prominence-i HPLC system (Shimadzu, Kioto, Japan) equipped with a quaternary pump, column oven, autosampler, and PDA detector. Separations were carried out on an Ascentis Express C18 column (3 µm, 100 × 4.6 mm; Supelco, Bellefonte, PA, USA) with a suitable guard column at 30 °C. The mobile phase consisted of 0.5% (*m/v*) orthophosphoric acid (solvent A) and acetonitrile (solvent B), both HPLC grade (Avantor Performance). The gradient program was: 0–1 min, 5% B in A; 1–30 min, 5–25% B; 30–31 min, 25–50% B; 31–35 min, 50% B; 35–36 min, 50–5% B; 36–42 min, 5% B. The flow rate was 1.4 mL/min. Approximately 20 mg of each extract was weighted and dissolved in methanol–water (7:3, *v*/*v*, 25 mL), filtered through a PTFE syringe filter (13 mm, 0.2 µm, Vitrum), and injected (5 µl). Quantification was performed using calibration curves constructed from appropriate authentic or in-house isolated compounds (Table S1, Supplementary Materials). Method validation (Tables S2–S3, Supplementary Materials) followed ICH guidelines^26^. Contents of the compounds were calculated per dry weight (DW) of the extracts. For each extract three technical replicates of the analysis were performed.

### Quantitative determination of proanthocyanidins

Total proanthocyanidins were quantified using the modified acid/butanol assay^27^. Aliquots of extract solutions (0.5 mL) were mixed with n-BuOH-35% HCl (95:5, *v/v*, 3 mL) and 2% (*w/v*) NH_4_Fe(SO_4_)_2_·12 H_2_O in 2 M HCl (0.1 mL). After 45 min incubation at 95.0 ± 0.2 °C, samples were cooled to 25 °C, and the absorbance was measured at 550 nm against the unheated sample used as a blank. Results were expressed as cyanidin chloride equivalents calculated using an external calibration curve (*n* = 7, *r* = 0.9999) prepared with cyanidin chloride as a reference standard (1.37–20.48 µg/mL). Results were calculated per DW of the extracts (mg CYE/g DW). For each extract three technical replicates of the analysis were performed.

### Antioxidant activity in non-cellular models

The scavenging capacity of the extracts towards O_2_^−^, OH˙, H_2_O_2_, and HClO was assessed in vitro using microplate spectrophotometric assays, following previously described procedures^28^. Ascorbic acid (AA) served as the positive control. SC_50_ values (concentration reducing oxidant levels by 50%) were determined from 5 to 10-point concentration–response curves and expressed in µg DW/mL and as mmol AA equivalents per DW of the extracts (mmol AAE/g DW). All measurements were performed in five independent technical replicates for each extract (*n* = 5).

### Antioxidant and anti-elastase activity in human neutrophils ex vivo

Buffy coat fractions were obtained from the Warsaw Blood Donation Centre. Donors were healthy, non-smoking males aged 18–35. The study adhered to the Declaration of Helsinki; no bioethics approval was required because only commercially available biological materials were used.

#### Isolation of human neutrophils

Neutrophils were isolated from the buffy coat fractions according to previously described method^29^ by dextran sedimentation, hypotonic erythrocyte lysis, and Ficoll-Hypaque gradient centrifugation. Cells were resuspended in (Ca^2+^)-free Hanks’ balanced salt solution (Sigma Aldrich, St. Louis, MO, USA), and maintained at 4 ℃ until use. The isolation was performed for each donor independently.

#### Neutrophils viability

Cytotoxicity of the extracts (50 μg/mL) and pure compounds (50 μM) was evaluated according to previously described method^30^ using propidium iodide (PI) staining and flow cytometry (BD FACSCalibur, BD Biosciences, San Jose, CA, USA). Triton X-100 solution served as the positive control. Measurements were performed after a 24-h incubation. Measurements were performed using neutrophils isolated form five independent donors (biological replicates), and each extract was tested in triplicate for each donor (technical replicates).

#### ROS generation by human neutrophils

Inhibition of ROS production was assessed using luminol-dependent chemiluminescence after stimulation with *N*-formyl-l-methionine-l-leucyl-l-phenylalanine (*f*MLP)^30^. Chemiluminescence was monitored in 96-well plates for 40 min at 2-min intervals using a Synergy 4 microplate reader (Biotech, Winooski, VT, USA). Results were expressed as percentage ROS secretion relative to extract-untreated control. Quercetin (1–50 μM) was employed as the positive control. Measurements were performed using neutrophils isolated form five independent donors (biological replicates), and each extract was tested in triplicate for each donor (technical replicates).

#### ELA-2 release by human neutrophils

Release of ELA-2 from *f*MLP-cytochalasin B-stimulated neutrophils was determined using *N*-succinyl-alanine-valine-*p*-nitroanilide (SAAVNA) as substrate, following previously described method^31^ Formation of *p*-nitrophenol was monitored at 412 nm for 300 min (20-min intervals) using the Synergy 4 reader. Results were expressed as percentage ELA-2 secretion relative to extract-untreated control. Quercetin (1–50 μM) was employed as a positive control. Measurements were performed using neutrophils isolated form five independent donors (biological replicates), and each extract was tested in triplicate for each donor (technical replicates).

### Statistical analysis

Results are presented as mean ± standard error (SE) from replicate measurements. Normality was verified using the Shapiro–Wilk test, and the homogeneity of variance using the Levene’s test. Statistical significance in the differences between the samples was evaluated using Welch’s ANOVA followed by the post-hoc Games-Howell’s test for multiple comparisons. Prior to multivariate analysis, the data were mean-centered and scaled to unit variance (autoscaling). No additional transformation was applied. PCA was performed using the correlation matrix. HCA was conducted using single linkage and Pearson’s *r* coefficient as the similarity metric. *Z*-scores were calculated according to the formula:

$$z= \frac{x-\mu }{\sigma }$$

where x denotes the mean activity of a given extract, µ is the mean activity of all extracts within a given assay, and σ is the corresponding standard deviation. Calculations were carried out in Statistica 13 Pl software (TIBCO Software Inc., Tulsa, OK, USA) and Microsoft Excel 2021 (Microsoft, Redmond, WA, USA) with the Real Statistics Resource Pack. Statistical significance was set at *p* < 0.05.

## Results

### Qualitative phytochemical analysis

The investigated extracts were initially subjected to UHPLC-PDA-ESI-MS^n^ profiling, that enabled the detection of 38 constituents, 35 of which were conclusively or tentatively identified as derivatives of flavonols, flavan-3-ols and phenolic acids (Table 2, Figure S1).

**Table 2:** UHPLC-PDA-ESI-MS/MS data of compounds detected in the investigated *Sambucus* extracts.

No	Rt (min)	UV λ_max_ (nm)	[M–H]^–^ (*m/z*)	Fragment ions (*m/z*)	Identification	Sample^g^											
						SCAE_L	SCAN_L	SKAM_F	SKAM_L	SNIG_F	SNIG_L	SRAC_F	SRAC_L	SSIB_F	SSIB_L	SWIL_F	SWIL_L
1	4.6	292	629	303	unidentified					(+)		+	+				
2	5.1	277	315	153	protocatechuic acid hexoside	+	(+)		++		(+)		++		+		+
3	6.1	272	203	–	unidentified	(+)	(+)	+	+	(+)	(+)	+	(+)	(+)	+	(+)	(+)
4	8.2	323	353	191, 179	3-*O*-caffeoylquinic acid (neochlorogenic acid)^a, b^	++	++	+	+	+	+	(+)	(+)	(+)	+	+	+
5	10.8	278	621	310	unidentified	+					+						
6	12.0	316	705	513, 321	caffeoylquinic acid dehydrodimer^c^	(+)	(+)	(+)	(+)	(+)	+	(+)	(+)	(+)	+	(+)	(+)
7	12.4	310	705	513, 321	caffeoylquinic acid dehydrodimer^c^	(+)	(+)	(+)	+	+	+	(+)	(+)	(+)	+	+	+
8	13.1	316	705	533, 513, 321	caffeoylquinic acid dehydrodimer^c^	+	(+)	(+)	(+)	(+)	(+)	(+)	(+)	(+)	+	(+)	(+)
9	13.7	278	451	289	epicatechin hexoside	(+)	(+)						(+)				
10	14.4	325	353	191	5-*O*-caffeoylquinic acid (chlorogenic acid)^a, b^	+++	+++	+++	+++	+++	+++	+++	+++	+++	+++	+++	+++
11	15.3	324	367	193	3-*O*-feruloylquinic acid^b^	(+)	+	+	+	(+)				(+)		(+)	+
12	16.0	324	353	173, 179, 191	4-*O*-caffeoylquinic acid (cryptochlorogenic acid)^a,b^	++	++	+	+	+	+	+	+	+	+	+	+
13	17.2	279	577	425, 407, 289	procyanidin dimer B-type	(+)	(+)										
14	18.4	279	577	425, 407, 289	procyanidin dimer B-type	(+)	(+)										
15	18.7	317	353	191	1-*O*-caffeoylquinic acid^e^	(+)	(+)	(+)	(+)	(+)	+	(+)	(+)	+	+	(+)	(+)
16	18.9	279	865	695, 713, 577	procyanidin trimer B-type		(+)										
17	20.2	279	289	245	(−)-epicatechin^a^	(+)	(+)										
18	20.4	316	337	191	5-*O*-*p*-coumaroylquinic acid^b^			(+)	(+)	+	(+)	(+)		(+)	(+)	+	(+)
19	20.8	312	337	173	4-*O*-*p*-coumaroylquinic acid^b^			(+)	(+)	(+)					(+)	(+)	+
20	23.1	324	367	191	5-*O*-feruloylquinic acid^b^	+	(+)	(+)	(+)	(+)	(+)	(+)	(+)	(+)	(+)	(+)	(+)
21	23.4	325	367	173	4-*O*-feruloylquinic acid^b^	(+)	(+)	(+)	+	(+)	(+)		(+)	(+)	(+)	(+)	+
22	26.6	255, 354	755	609, 591, 489, 301	quercetin di-deoxyhexoside-hexoside^f^					(+)	+						
23	27.0	255, 354	609	447, 463, 301	quercetin hexoside-deoxyhexoside							+				++	
24	31.4	265, 360	609	301	quercetin 3-*O*-rutinoside (rutin)^a^	(+)	+++	+++	++	+++	+++	(+)	+	(+)	(+)	+++	+++
25	31.4	254, 353	623	477, 461, 315	isorhamnetin hexoside-deoxyhexoside							+					+
26	32.5	255, 354	463	301	quercetin 3-*O*-β-d-glucopyranoside (isoquercitrin)^a^	++	(+)	+	(+)	++	(+)	+	+	+++	++	+	+
27	35.2	254, 354	665	519, 461, 315	isorhamnetin acetylhexoside-deoxyhexoside							+					
28	35.3	267, 351	447	285	kaempferol hexoside	+											
29	35.8	267, 357	593	285	kaempferol 3-*O*-rutinoside^a^	(+)	+++	+	++	+	+		+			(+)	(+)
30	36.2	255, 354	505	463, 301	quercetin acetylhexoside	+				+		+	+	++	+		
31	36.6	254, 355	623	315	isorhamnetin 3-*O*-rutinoside^a^	(+)	(+)	++		++	(+)	+	(+)			+	
32	36.8	264, 351	447	285	kaempferol 3-*O*-β-d-glucopyranoside (astragalin)^a^	++	(+)	(+)		+	(+)	(+)	+	+	++		(+)
33	37.8	325	515	353 − > 191, 179 (MS^3^)	3,5-di-*O*-caffeoylquinic acid^b^	(+)		++	+	++	+	+	+	++	+	++	+
34	37.9	258, 354	477	315	isorhamnetin 3-*O*-β-d-glucopyranoside^a^	++		+		+	(+)	+	(+)	+		(+)	
35	41.1	325	515	353 − > 173 (MS^3^)	4,5-di-*O*-caffeoylquinic acid^b^			+	(+)	+	(+)	+	(+)	+	(+)	+	(+)
36	41.5	256, 349	489	285	kaempferol acetylhexoside	+				(+)		(+)	+	(+)	+		
37	42.8	256, 355	519	315	isorhamnetin acetylhexoside	+				+		+	(+)	+	(+)		
38	52.6	288	271	151	naringenin			+		+		+		+		+	

R_t,_ retention time. UV λ_max_, absorbance maxima in PDA spectra. [M–H]^–^, deprotonated molecule. Refer to Table 1 for extract abbreviations. ^a^ identified with reference standard; ^b^^32^; ^c^^33^; ^d^^34^; ^e^^35^; ^f^^13^; ^g^ estimated abundance based on the relative peak intensity at λ = 280 nm compared to the most intense peak in the extract: (+), < 4%; +, 4–15%; +  +, 15–60%; +  +  +, > 60%.

Among the detected compounds, flavonols were the most numerous, with 15 representatives identified (22–32, 34, 36–38). This group comprised glycosides of quercetin, kaempferol and isorhamnetin containing from one to three sugar moieties. Quercetin 3-*O*-rutinoside (24) and quercetin 3-*O*-β-d-glucopyranoside (26) were present in all extracts, although in some cases only at trace levels. Other relatively ubiquitous flavonoids were kaempferol and isorhamnetin 3-*O*-β-d-glucosides and 3-*O*-rutinosides (29, 31, 32, 34), which occurred in detectable amounts in 8–10 of 12 extracts. By contrast, several flavonols were characteristic of only one or two extracts. For example, quercetin di-deoxyhexoside-hexoside (22) was detected exclusively in *S. nigra* extracts, whereas the presence of minor amounts of compounds 23, 25 and 27 distinguished the flower extract of *S. racemosa* from all others.

The second major group of constituents (4, 6–8, 10–12, 15, 18–21, 33, 35) comprised phenolic acid derivatives, primarily quinic acid pseudodepsides. The predominant constituent within this group was 5-*O*-caffeoylquinic acid (10) that occurred in high amounts in all the extracts. Its isomers, 3-*O*-caffeoylquinic acid (4), 4-*O*-caffeoylquinic acid (12), and 1-*O*-caffeoylquinic acid (15), were also detected in every sample. Two dicaffeoylquinic acids (33, 35) were present in most of the extracts except for those of *S. canadensis* and *S. caerulea* leaves. Additionally, smaller quantities of other pseudodepsides were identified, including isomers of *p*-coumaroylquinic (18, 19) and feruloylquinic acids (11, 20, 21). All extracts contained also minor amounts of caffeoylquinic acid oxidation products, identified as caffeoylquinic acid dehydrodimers (6–8).

Flavan-3-ols derivatives (9, 13, 14, 16, 17) were detected only in trace amounts and essentially only in the leaf extracts of *S. canadensis* and S. *caerulea*. They included (−)-epicatechin (19), two procyanidin type B dimers (13, 15), one type B trimer (18) and an epicatechin hexoside (9).

No signals matching the retention and characteristic fragmentation pattern of sambunigrin reference standard were observed in the investigated extracts under the applied LC–MS conditions.

### Quantitative HPLC-PDA analysis

Quantitative determination was performed for 24 constituents present at measurable concentration in at least one extract. Accurate quantification was achieved for seven compounds for which authentic standards were available (5-*O*-caffeoylquinic acid, quercetin 3-*O*-β-d-glucopyranoside, quercetin 3-*O*-rutinoside, kaempferol 3-*O*-β-d-glucopyranoside, kaempferol-3-*O*-rutinoside, isorhamnetin 3-*O*-β-d-glucopyranoside, and isorhamnetin 3-*O*-rutinoside). The remaining analytes were quantified using calibration curves constructed for structurally related standards.

**Fig. 1:**
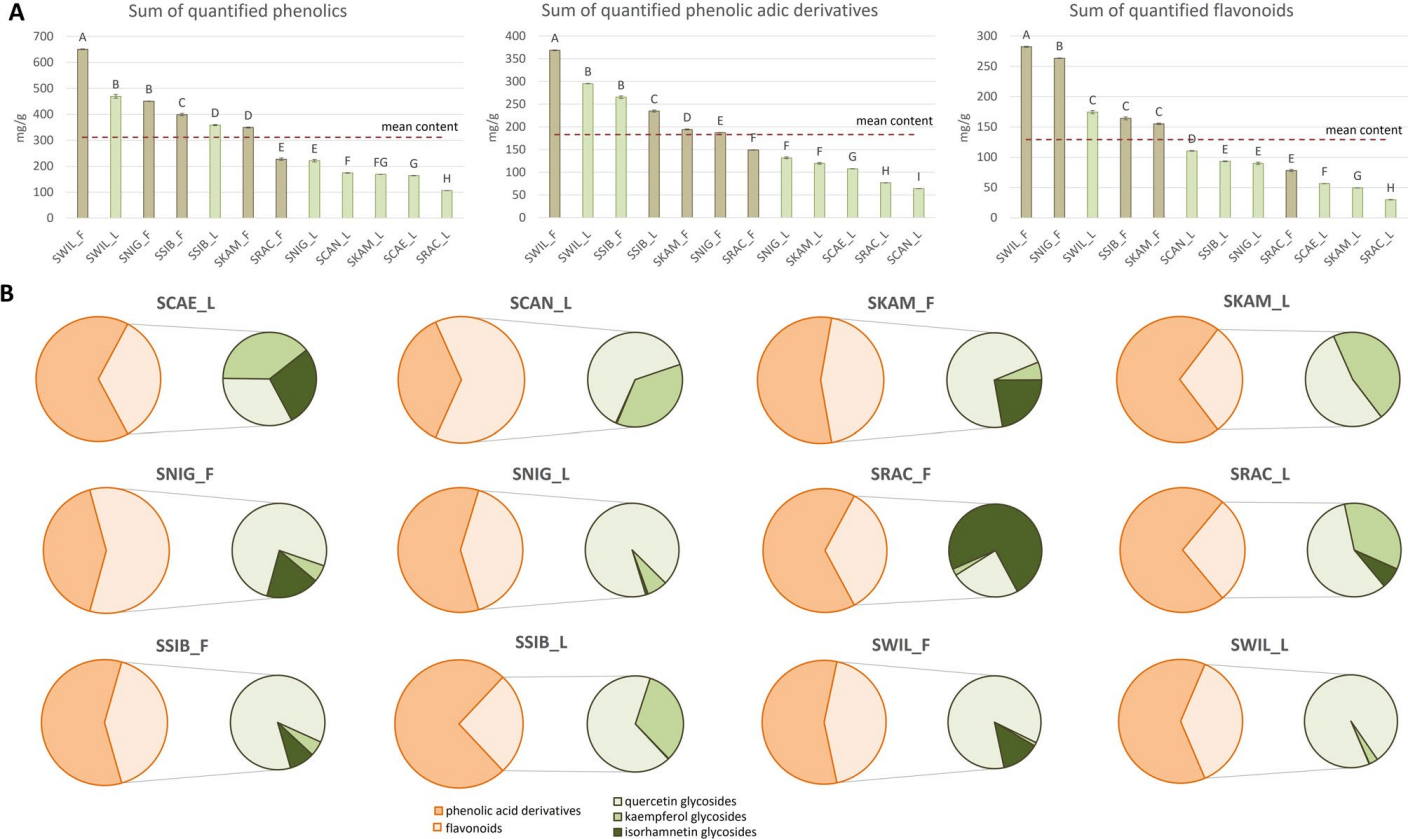
Results of quantitative analysis of phenolics in the investigated *Sambucus* extracts. (**A**) Extracts ranked according to the content of main group of phenolics. Results presented as means ± SE (*n* = 3). The statistical differences between means were assessed using Welch’s ANOVA followed by Games-Howell’s post-hoc test. Means sharing the same capital letter for a given analysis are not significantly different at *α* = 0.05; (**B**) Relative proportions between the contents of the main groups of phenolics in the investigated extracts. For extract abbreviation refer to Table 1.

The sum of quantified phenolic compounds varied markedly among the samples, ranging from 106.24 mg/g in the *S. racemosa* leaf extract to 650.82 mg/g in the *S. williamsii* flower extract. In all cases, flower extracts (227.04–650.82 mg/g) contained higher levels of phenolics than the corresponding leaf extracts (106.24–469.33 mg/g). Notably, *S. williamsii* and *S. sibirica* exhibited above average polyphenol concentrations in both organs. The *S. nigra* flower extract ranked second in terms of sum of quantified polyphenols (450.41 mg/g), but its leaf extract, along with majority of other leaf samples, was among the least enriched in phenolics (Table 3, Fig. 1A).

**Table 3:** Content (in mg/g DW) of the individual analytes in the investigated extracts of *Sambucus* species.

Analyte	Extract											
	SCAE_L	SCAN_L	SKAM_F	SKAM_L	SNIG_F	SNIG_L	SRAC_F	SRAC_L	SSIB_F	SSIB_L	SWIL_F	SWIL_L
3-CQA^b^ (4)	14.03 ± 0.06^A^	12.59 ± 0.06^B^	7.30 ± 0.07^E^	9.39 ± 0.01^D^	10.76 ± 0.07^C^	7.89 ± 0.12^E^	1.03 ± 0.03^I^	2.26 ± 0.01^H^	2.88 ± 0.03^G^	8.78 ± 0.07^D^	6.04 ± 0.03^F^	16.15 ± 0.28^A^
5-CQA^a^ (10)	75.44 ± 0.84^F^	38.33 ± 0.34^H^	123.27 ± 0.44^D^	75.21 ± 0.27^F^	93.19 ± 0.63^E^	100.17 ± 3.38^E^	114.38 ± 4.05^DE^	59.36 ± 0.20^G^	182.37 ± 2.26^C^	217.29 ± 1.51^B^	301.23 ± 0.96^A^	234.76 ± 6.42^B^
3-FQA^b^ (11)	0.68 ± 0.01^F^	1.81 ± 0.01^D^	3.64 ± 0.03^B^	7.72 ± 0.04^A^	0.85 ± 0.01^E^	n.d	n.d	n.d	0.24 ± 0.01^G^	n.d	2.14 ± 0.02^C^	7.04 ± 0.12^A^
4-CQA^b^ (12)	12.10 ± 0.23^B^	7.65 ± 0.06^DE^	4.77 ± 0.08^H^	10.26 ± 0.08^C^	6.15 ± 0.04^F^	8.42 ± 0.33^D^	4.92 ± 0.17^GH^	3.87 ± 0.03^I^	7.31 ± 0.10^E^	13.20 ± 0.11^A^	5.64 ± 0.03^G^	11.76 ± 0.33^ABC^
1-CQA^b^ (15)	< LOQ	1.51 ± 0.02^DE^	0.54 ± 0.01^G^	0.91 ± 0.04^F^	2.48 ± 0.02^C^	7.46 ± 0.13^A^	1.35 ± 0.03^E^	1.58 ± 0.02^D^	3.01 ± 0.14^C^	4.67 ± 0.02^B^	< LOQ	< LOQ
5-FQA^b^ (18)	2.98 ± 0.02^A^	< LOQ	0.38 ± 0.02^H^	1.19 ± 0.01^CD^	0.76 ± 0.01^E^	1.26 ± 0.03^C^	0.31 ± 0.01^H^	0.70 ± 0.01^F^	0.79 ± 0.01^F^	2.76 ± 0.02^B^	0.62 ± 0.01^G^	1.01 ± 0.02^D^
4-FQA^b^ (19)	1.21 ± 0.01^DF^	1.16 ± 0.01^E^	1.78 ± 0.03^C^	5.43 ± 0.01^A^	< LOQ	1.37 ± 0.04^DE^	n.d	1.02 ± 0.05^FG^	< LOQ	0.86 ± 0.02^G^	< LOQ	3.69 ± 0.09^B^
5-*p*CQA^b^ (20)	n.d	n.d	0.92 ± 0.01^D^	0.60 ± 0.03^E^	3.69 ± 0.02^B^	< LOQ	1.37 ± 0.03^C^	n.d	< LOQ	0.74 ± 0.01^E^	5.18 ± 0.03^A^	0.88 ± 0.02^D^
4-*p*CQA^b^ (21)	n.d	n.d	0.70 ± 0.01^E^	1.58 ± 0.02^B^	0.85 ± 0.01^D^	n.d	n.d	n.d	n.d	1.03 ± 0.01^C^	1.06 ± 0.01^C^	4.75 ± 0.08^A^
3.5-DCQA^b^ (33)	1.14 ± 0.02^I^	n.d	43.52 ± 0.19^B^	5.46 ± 0.01^G^	55.66 ± 0.18^A^	2.97 ± 0.7^H^	19.24 ± 0.33^E^	5.01 ± 0.04^G^	32.55 ± 0.31^D^	13.26 ± 0.10^F^	40.76 ± 0.29^C^	12.46 ± 0.22^F^
4.5-DCQA^b^ (35)	n.d	n.d	7.29 ± 0.05^B^	1.56 ± 0.01^F^	12.52 ± 0.04^A^	0.74 ± 0.01^G^	6.51 ± 0.18^BCD^	1.25 ± 0.04^F^	5.45 ± 0.05^D^	2.32 ± 0.02^E^	5.89 ± 0.03^C^	2.38 ± 0.05^E^
**Sum of phenolic acid pseudodepsides**	**107.57 ± 1.24**^**G**^	**63.75 ± 0.56**^**I**^	**194.09 ± 1.13**^**D**^	**119.30 ± 0.70**^**F**^	**186.92 ± 0.93**^**E**^	**131.63 ± 4.47**^**F**^	**149.12 ± 5.24**^**F**^	**76.56 ± 0.07**^**H**^	**234.59 ± 4.63**^**C**^	**265.18 ± 3.22**^**B**^	**368.54 ± 1.45**^**A**^	**294.87 ± 8.20**^**B**^
QTG^c^ (22)	n.d	n.d	n.d	n.d	1.32 ± 0.03^B^	9.10 ± 0.21^A^	n.d	n.d	n.d	n.d	n.d	n.d
QDG^c^ (23)	n.d	n.d	n.d	n.d	n.d	n.d	3.74 ± 0.08^B^	n.d	n.d	n.d	57.89 ± 0.28^A^	n.d
QRT^a^ (24)	1.03 ± 0.04^H^	68.82 ± 0.35^D^	96.84 ± 0.82^C^	25.57 ± 0.05^E^	153.51 ± 0.19^B^	72.08 ± 1.56^D^	3.01 ± 0.07^G^	6.84 ± 0.15^F^	0.57 ± 0.02^I^	0.25 ± 0.01^ J^	180.17 ± 0.51^A^	153.22 ± 2.32^B^
QG^a^ (26)	13.18 ± 0.15^E^	0.82 ± 0.01^ J^	14.17 ± 0.11^DE^	0.99 ± 0.01^I^	34.48 ± 0.08^C^	1.65 ± 0.05^H^	4.22 ± 0.15^FG^	5.33 ± 0.04^FG^	87.68 ± 1.69^A^	41.56 ± 0.24^B^	3.48 ± 0.05^G^	15.63 ± 0.23^D^
QAH^d^ (30)	4.38 ± 0.01^F^	n.d	n.d	n.d	10.99 ± 0.03^C^	n.d	7.67 ± 0.17^D^	4.95 ± 0.03^E^	53.20 ± 0.64^A^	20.74 ± 0.18^B^	n.d	n.d
**Sum of quercetin glycosides**	**18.59 ± 0.19**^** J**^	**69.63 ± 0.35**^**G**^	**111.01 ± 1.61**^**E**^	**26.56 ± 0.04**^**I**^	**200.29 ± 0.45**^**B**^	**82.83 ± 3.14**^**F**^	**18.64 ± 0.78**^**JK**^	**17.12 ± 0.20**^** K**^	**141.45 ± 2.22**^**D**^	**62.47 ± 0.33**^**H**^	**241.54 ± 1.37**^**A**^	**168.85 ± 2.55**^**C**^
KRT^a^ (29)	4.69 ± 0.02^F^	39.86 ± 0.21^A^	7.89 ± 0.04^D^	22.89 ± 0.01^B^	8.23 ± 0.03^C^	6.16 ± 0.09^E^	n.d	3.38 ± 0.05^G^	n.d	n.d	3.47 ± 0.01^G^	4.85 ± 0.09^F^
KG^a^ (32)	13.70 ± 0.07^B^	0.44 ± 0.01^G^	1.62 ± 0.01^E^	n.d	6.33 ± 0.02^C^	n.d	1.03 ± 0.04^F^	3.74 ± 0.08^D^	5.62 ± 0.12^C^	21.26 ± 0.01^A^	n.d	0.50 ± 0.01^G^
KAH^e^ (36)	3.69 ± 0.01^B^	n.d	n.d	n.d	0.76 ± 0.01^D^	n.d	0.78 ± 0.02^D^	3.26 ± 0.01^C^	2.82 ± 0.36^C^	9.32 ± 0.09^A^	n.d	n.d
**Sum of kaempferol glycosides**	**22.08 ± 0.16**^**C**^	**40.30 ± 0.38**^**A**^	**9.52 ± 0.08**^**E**^	**22.88 ± 0.01**^**C**^	**15.33 ± 0.07**^**D**^	**6.16 ± 0.16**^**F**^	**1.81 ± 0.10**^**I**^	**10.38 ± 0.2**^**E**^	**8.45 ± 0.67**^**E**^	**30.58 ± 0.37**^**B**^	**3.47 ± 0.01**^**H**^	**5.35 ± 0.17**^**G**^
IDG^f^ (25)	n.d	n.d	n.d	n.d	n.d	n.d	21.55 ± 0.50	n.d	n.d	n.d	16.95 ± 0.05	n.d
IADG^f^ (27)	n.d	n.d	n.d	n.d	n.d	n.d	10.37 ± 0.26	n.d	n.d	n.d	n.d	n.d
IRT^a^ (31)	0.89 ± 0.01^D^	0.44 ± 0.03^EF^	25.72 ± 0.18^A^	n.d	25.85 ± 0.12^A^	0.51 ± 0.01^E^	5.10 ± 0.11^C^	0.94 ± 0.02^D^	n.d	n.d	19.28 ± 0.05^B^	n.d
IG^a^ (34)	11.85 ± 0.06^B^	n.d	8.83 ± 0.04^C^	n.d	18.98 ± 0.07^A^	0.24 ± 0.01^F^	12.94 ± 0.04^B^	0.71 ± 0.02^E^	9.19 ± 0.03^C^	n.d	1.03 ± 0.03^D^	n.d
IAH^g^ (37)	2.90 ± 0.02^C^	n.d	n.d	n.d	3.03 ± 0.01^C^	n.d	7.48 ± 0.12^A^	0.53 ± 0.01^D^	5.01 ± 0.09^B^	< LOQ	n.d	n.d
**Sum of isorhamnetin glycosides**	**15.65 ± 0.12**^**D**^	**0.44 ± 0.06**^**FG**^	**34.55 ± 0.22**^**C**^	**n.d**	**47.86 ± 0.25**^**A**^	**0.75 ± 0.01**^**F**^	**57.46 ± 2.27**^**A**^	**2.18 ± 0.05**^**E**^	**14.21 ± 0.25**^**D**^	** < LOQ**	**37.27 ± 0.20**^B^	**n.d**
**Sum of flavonoids**	**56.32 ± 0.23**^**F**^	**110.38 ± 0.59**^**D**^	**155.08 ± 2.07**^**C**^	**49.44 ± 0.05**^**G**^	**263.48 ± 0.74**^**B**^	**89.90 ± 3.32**^**E**^	**77.92 ± 3.14**^**E**^	**29.67 ± 0.42**^**H**^	**164.12 ± 4.11**^**C**^	**93.25 ± 0.54**^**E**^	**282.27 ± 1.55**^**A**^	**174.46 ± 4.60**^**C**^
**Sum of phenolics**	**163.90 ± 0.85**^**G**^	**174.11 ± 0.87**^**F**^	**349.17 ± 1.82**^**D**^	**168.74 ± 0.75**^**FG**^	**450.41 ± 1.39**^**B**^	**221.53 ± 4.07**^**E**^	**227.04 ± 3.75**^**E**^	**106.24 ± 0.41**^**H**^	**398.69 ± 4.70**^**C**^	**358.43 ± 2.32**^**D**^	**650.82 ± 1.44**^**A**^	**469.33 ± 8.48**^**B**^

Results presented as means ± SE (*n* = 3). LOQ, limit of quantitation. CQA, caffeoylquinic acid; DCQA, dicaffeoylquinic acid; FQA, feruloylquinic acid; IADG, isorhamnetin acetyldiglycoside; IAH, isorhamnetin acetylhexoside; IDG, isorhamnetin diglycoside; IG, isorhamnetin 3-*O*-β-d-glucopyranoside; IRT, isorhamnetin 3-*O*-rutinoside; KAH, kaempferol acetylhexoside; KG, kaempferol 3-*O*-β-d-glucopyranoside; KRT, kaempferol 3-*O*-rutinoside; *p*CQA, *p*-coumaroylquinic acid; QAH, quercetin acetylhexoside; QDG, quercetin diglycoside; QG, quercetin 3-*O*-β-d-glucopyranoside; QRT, quercetin 3-*O*-rutinoside; QTG, quercetin triglycoside. The statistical differences between means were assessed using Welch’s ANOVA followed by Games-Howell’s post-hoc test. Means sharing the same capital letter for a given constituent are not significantly different at *α* = 0.05. ^a^quantified using an authentic reference standard; ^b^quantified using the calibration curve of 3-CQA; ^c^quantified using the calibration curve of QRT; ^d^quantified using the calibration curve of QG; ^e^quantified using the calibration curve of KG; ^f^quantified using the calibration curve of IRT; ^g^quantified using the calibration curve of IG. Bolded rows represent the sum of the analytes within the respective group.

Phenolic acid pseudodepsides were the dominant class (63.75–368.54 mg/g) in most extracts, with the exception of the *S. nigra* flower and the *S. canadensis* leaf extracts, where flavonoids prevailed (Table 3, Fig. 1B). The highest levels of pseudodepsides were present in the flower and leaf extracts of *S. williamsii* and *S. sibirica*, while their content in the extract of *S. nigra* flowers was only moderate. 5-*O*-Caffeoylquinic acid was present in substantial amounts in all samples (38.33–301.23 mg/g), consistently being the major pseudodepside and, in most cases, the most abundant polyphenol overall. Moreover, 3,5-di-*O*-caffeoylquinic acid was found in notable quantities in all flower extracts (19.24–55.66 mg/g), but occurred at much lower levels in the leaf samples (0–13.26 mg/g).

The sum of quantified flavonoids ranged from 27.39 mg/g in *S. racemosa* leaf extract to 286.97 mg/g in *S. williamsii* flower extract (Table 3, Fig. 1A). *S. nigra* flower extract was also particularly flavonoid-abundant (263.48 mg/g), while the *S. williamsii* leaf extract, which ranked third, contained over 30% less of flavonoids (174.46 mg/g). In most samples, quercetin derivatives dominated. Exceptions included *S. caerulea* leaf extract, in which kaempferol derivatives slightly exceeded quercetin analogues, and the *S. racemosa* flower extract, where isorhamnetin derivatives prevailed (Table 3, Fig. 1B). Among individual flavonols, quercetin 3-*O*-rutinoside was the principal compound in seven of the twelve extracts, including those from *S. canadensis*, *S. williamsii*, *S. nigra* and *S. kamtschatica* (25.57–180.17 mg/g). In contrast, *S. sibirica* extracts contained only small amounts of quercetin 3-*O*-rutinoside but were rich in quercetin 3-*O*-β-d-glucopyranoside (41.56–87.68 mg/g) and acetylated quercetin hexoside derivative (20.74–53.20 mg/g).

### Condensed proanthocyanidin content

In addition to low-molecular-weight polyphenols detectable by RP-HPLC, plant materials may also contain high-molecular-weight polyphenolic compounds, such as oligomeric and polymeric proanthocyanidins, which can contribute to the overall biological activity of the extracts. Their content was therefore assessed using a dedicated spectrophotometric assay.

Overall, *Sambucus* extracts contained relatively low levels of condensed flavan-3-ols (Figure S2). Proanthocyanidins were not detected in either flower or leaf extracts of *S. nigra*. Similarly, no proanthocyanidins were observed in the leaf extracts of *S. kamtschatica*, *S. sibirica*, and *S. williamsii*, although their corresponding flower extracts contained detectable amounts of these compounds (6.74–8.16 mg CYE/g). In contrast, *S. racemosa* exhibited low proanthocyanidin levels in both flowers and leaves (4.59–4.85 mg CYE/g). The highest concentrations of condensed flavan-3-ols were found in the leaf extracts of *S. caerulea* and *S. canadensis* (11.05–16.03 mg CYE/g).

### Principal component analysis

**Fig. 2:**
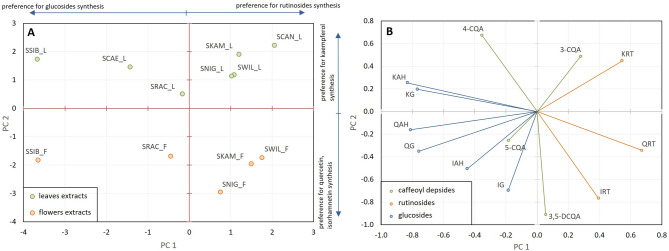
Results of the PCA analysis. (**A**) PC 1 vs PC 2 score plot for the investigated samples. (**B**) PC 1 vs PC 2 loading plot for the investigated variables. Refer to Table 1 for extracts abbreviations. CQA, caffeoylquinic acid; DCQA, dicaffeoylquinic acid; IAH, isorhamnetin acetylhexoside; IG, isorhamnetin 3-*O*-β-d-glucopyranoside; IRT, isorhamnetin 3-*O*-rutinoside; KAH, kaempferol acetylhexoside; KG, kaempferol 3-*O*-β-d-glucopyranoside; KR, kaempferol 3-*O*-rutinoside; QAH, quercetin acetylhexoside; QG, quercetin 3-*O*-β-d-glucopyranoside; QRT, quercetin 3-*O*-rutinoside.

To gain deeper insight into the compositional variation across samples, PCA was performed using quantitative data for the thirteen most abundant constituents (Fig. 2). These included major pseudodepsides (3-*O*-caffeoylquinic, 4-*O*-caffeoylquinic, 5-*O*-caffeoylquinic, and 3,5-dicaffeoylquinic acids) as well as key flavonoids (rutinosides, glucosides and acetylhexosides of kaempferol, quercetin and isorhamnetin).

Principal Component 1 (PC1) accounted for 29% of the total variance. Interestingly, flower and leaf extracts from the same species exhibited remarkably similar PC1 scores, indicating that species identity had a stronger influence on PC1 than plant organ. Correspondingly, extract distribution along the PC1 axis largely followed species-specific pattern (Fig. 2A). Variable loadings showed that the PC1 was strongly positively associated with quercetin 3-*O*-rutinoside content and, to a lesser extent, with kaempferol and isorhamnetin rutinosides. In contrast, glucosides and acetylhexosides (especially those of kaempferol and quercetin) contributed negatively to PC1 (Fig. 2B). Thus, PC1 effectively reflected the relative tendency of a species to accumulate rutinosides (positive score) versus glucosides (negative score). This trend was visible in the score plot: the extracts positioned on the right were dominated by quercetin 3-*O*-rutinoside, while *S. sibirica* extracts, located at the far left with the lowest PC1 score, exhibited negligible levels of rutinoside and a strong predominance of quercetin 3-*O*-glucopyranoside (Fig. 2A).

Principal Component 2 (PC2) explained 20% of the total variance and separated flower and leaf extracts, with leaf samples scoring positively and the flower samples negatively (Fig. 2A). Quinic acid pseudodepsides contributed the most to PC2 variation. Specifically, 3,5-dicaffeoylquinic acid was strongly negatively associated with PC2, whereas 4-*O*-caffeoylquinic and 3-*O*-caffeoylquinic acids showed positive PC2 loadings. Flavonoid composition also influenced PC2, though to a lesser extent (Fig. 2B). Extracts richer in isorhamnetin and quercetin glycosides tended to display lower PC2 scores, while those containing higher proportion of kaempferol derivatives exhibited more positive PC 2 values. Quantitative data supported this pattern; although leaves generally contained higher absolute levels of quercetin derivatives, the quercetin-to-kaempferol ratio was generally lower in leaves than in flowers of the same species.

**Fig. 3:**
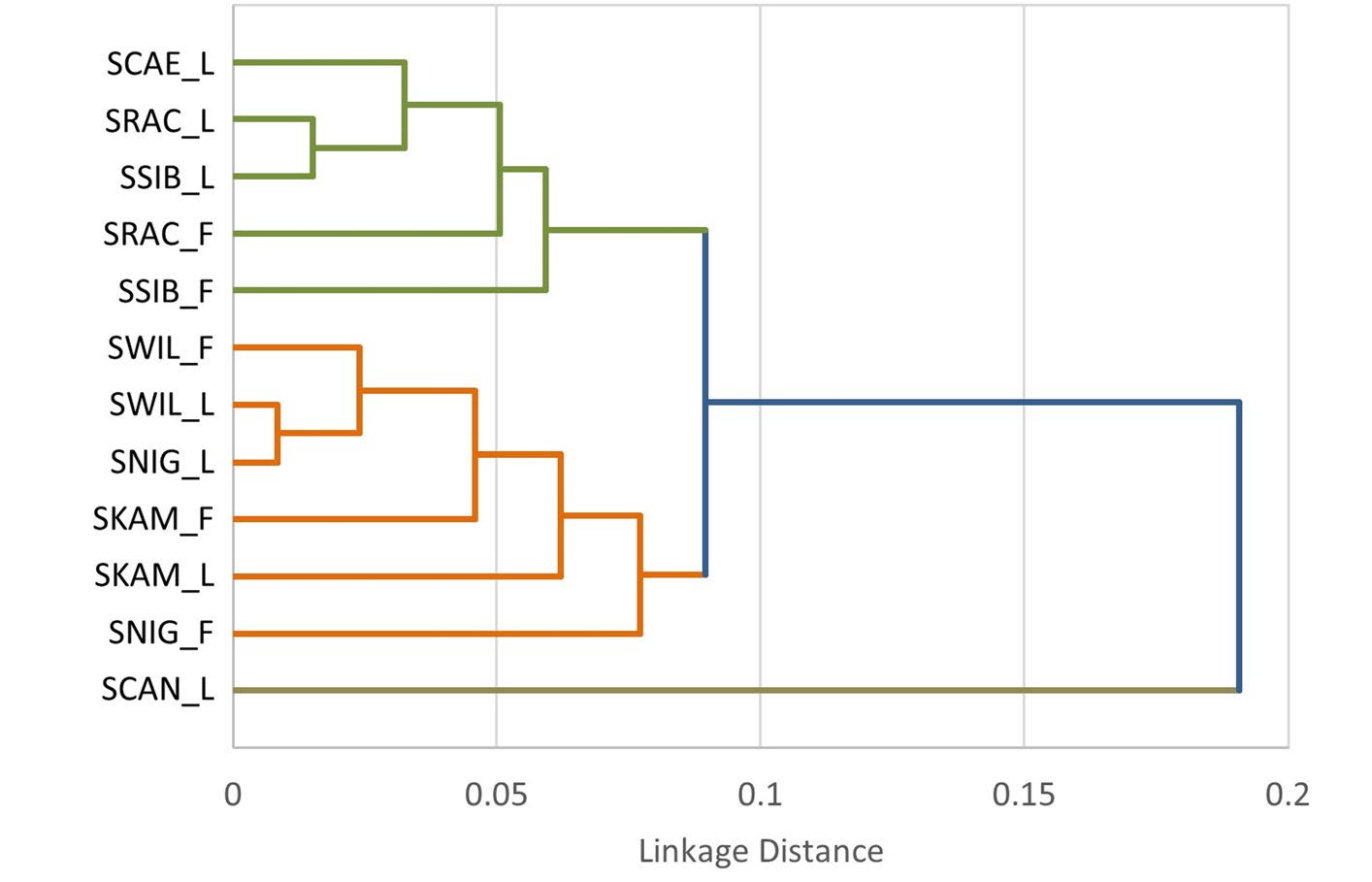
Dendrogram resulting from Hierarchical Cluster Analysis (HCA) of polyphenol levels in the studied *Sambucus* extracts. The analysis employed single linkage and Pearson’s *r* coefficient as the similarity metric. The vertical axis represents the distance at which clusters were merged. Three distinct clusters were distinguished, highlighting differences in phenolic composition. Refer to Table 1 for extracts abbreviations.

To further explore the relationships among samples, the same data matrix was subjected to HCA, what resulted in the identification of three distinct clusters (Fig. 3).

Cluster I included extracts from *S. nigra*, *S. kamtschatica* and *S. williamsii*. These samples were positioned on the right side of the PCA score plot and were characterized by high levels of quercetin 3-*O*-rutinoside and 5-*O*-caffeoylquinic acid. Cluster II comprised extracts from *S. caerulea*, *S. racemosa* and *S. sibirica*. Although 5-*O*-caffeoylquinic acid remained the predominant pseudodepside in this group, quercetin 3-*O*-rutinoside was present only in very small amounts. The extracts in this cluster showed negative PC1 scores and generally low abundance of flavonol rutinosides, with glucosides representing the dominant flavonoid for each aglycone. Moreover, all extracts in Cluster II contained acetylated flavonol derivatives, in contrast to Cluster I where only *S. nigra* flower extract showed limited acetylhexoside presence. Cluster III consisted exclusively of the *S. canadensis* leaf extract. This sample shared some similarities with Cluster I (notably, high quercetin 3-*O*-rutinoside content) but formed a separate cluster likely due to its relatively low level of 5-*O*-caffeoylquinic acid and comparatively high concentrations of kaempferol 3-*O*-rutinoside.

### Antioxidant activity in chemical models

As an initial assessment of a biological potential of the extracts, we evaluated their scavenging capacity against several physiologically relevant ROS including H_2_O_2_, OH˙, O_2_^−^, HClO. All extracts demonstrated concentration-dependent quenching activity; however, relevant differences were observed in both the potency of individual extracts and their specificity toward particular ROS (Table 4, Figure S3).

**Table 4:** Scavenging effects of the investigated *Sambucus* extracts towards selected reactive oxygen species.

Analyte	H_2_O_2_		OH˙		O_2_^−^		HClO		Mean *z*-score
	SC_50_ [µg/mL]	*z*-score^a^	SC_50_ [µg/mL]	*z*-score^a^	SC_50_ [µg/mL]	*z*-score^a^	SC_50_ [µg/mL]	*z*-score^a^	
SSIB_F	51.4 ± 1.4^C^	− 1.09	76.4 ± 2.3^A^	− 0.94	11.4 ± 0.6^C^	− 0.75	7.3 ± 0.2^A^	− 1.22	− 1.00
SWIL_F	65.0 ± 0.7^D^	− 0.70	72.9 ± 3.8^A^	− 1.03	14.0 ± 0.5^CD^	− 0.11	7.9 ± 0.3^A^	− 1.10	− 0.74
SSIB_L	75.2 ± 0.8^E^	− 0.40	84.6 ± 2.1^A^	− 0.73	8.4 ± 0.8^B^	− 1.47	14.5 ± 0.6^C^	0.05	− 0.64
SWIL_L	34.9 ± 1.5^B^	− 1.57	130.1 ± 6.1^BCD^	0.45	13.8 ± 0.3^CD^	− 0.17	11.7 ± 0.3^B^	− 0.43	− 0.43
SKAM_F	87.9 ± 1.6^F^	− 0.03	70.2 ± 2.5^A^	− 1.10	16.9 ± 1.9^D^	0.59	9.0 ± 0.3^A^	− 0.91	− 0.36
SRAC_F	80.0 ± 1.3^EF^	− 0.26	83.5 ± 2.5^A^	− 0.76	17.0 ± 1.6^DE^	0.62	11.8 ± 0.5^BC^	− 0.41	− 0.20
SKAM_L	67.8 ± 0.7^D^	− 0.61	149.9 ± 9.1^D^	0.96	16.2 ± 1.1^DE^	0.42	14.5 ± 0.4^BC^	− 0.30	0.12
SCAN_L	100.1 ± 2.3^G^	0.33	195.9 ± 6.5^E^	2.17	8.9 ± 0.6^B^	− 1.36	13.9 ± 0.7^BC^	− 0.05	0.27
SNIG_F	110.4 ± 2.8^G^	0.63	110.3 ± 4.2^B^	− 0.06	22.3 ± 1.9^E^	1.94	13.5 ± 0.5^BC^	− 0.12	0.60
SNIG_L	100.9 ± 5.3^FG^	0.35	111.5 ± 4.1^BC^	− 0.03	18.8 ± 1.7^E^	1.08	20.3 ± 0.2^D^	1.07	0.62
SRAC_L	146.1 ± 5.6^H^	1.67	117.6 ± 6.3^BCD^	0.13	11.4 ± 0.7^C^	− 0.75	24.4 ± 1.1^D^	1.80	0.71
SCAE_L	146.9 ± 4.9^H^	1.69	148.2 ± 5.2^D^	0.92	14.3 ± 0.7^CD^	− 0.03	23.4 ± 0.2^D^	1.62	1.05
AA	17.8 ± 0.4^A^		141.1 ± 4.4^CD^		4.4 ± 0.5^A^		7.5 ± 0.2^A^		

Results presented as means ± SE (*n* = 5). The statistical differences between means were assessed using Welch’s ANOVA followed by Games-Howell’s post-hoc test. Means sharing the same capital letter for a given radical are not significantly different at *α* = 0.05. Refer to Table 1 for extracts abbreviations; AA, ascorbic acid (positive control). ^a^The *z*-scores were calculated from SC_50_ values, lower *z*-scores correspond to stronger scavenging activity, higher *z*-scores indicate weaker activity.

When expressed relative to ascorbic acid, the extracts exhibited the greatest efficiency against OH˙. With the exception of the *S. canadensis* leaf extract (4.11 mmol AA/g), all extracts showed activity comparable to or exceeding that of AA (5.28 mmol AA/g), ranging from 5.43 mmol AA/g to 11.48 mmol AA/g (Figure S3A). Similarly, in the HClO scavenging assay (Figure S3B), the most active extracts, from the flowers of *S. sibirica* (5.73 mmol AA/g) and flowers of *S. williamsii* (5.36 mmol AA/g), displayed efficacy equivalent to AA (5.28 mmol AA/g). In both assays, flower extracts were generally at least as effective as the corresponding leaf extracts, likely reflecting their higher phenolic content.

In contrast, activity against O_2_^−^ and H_2_O_2_ was overall weaker, with the most potent extracts achieving approximately half the activity of AA (5.28 mmol AA/g). For H_2_O_2_ scavenging, the most active was the *S. williamsii* leaf extract (2.67 mmol AA/g). Against O_2_^−^ the most effective were leaf extracts of *S. sibirica* (2.99 mmol AA/g) and *S. canadensis* (2.78 mmol AA/g). Interestingly, in these assays leaf extracts often outperform their flower counterparts, suggesting significant contribution of leaf-specific phenolic or non-phenolic constituents to their ROS-neutralizing selectivity (Figure S3CD).

To obtain an integrated measure of antioxidant performance, *z*-scores were calculated for each extract across all four assays, and mean *z*-scores were used as indicators of overall activity (Table 4). As *z*-scores were calculated using SC_50_ value the lowest *z*-score values identified flower and leaf extracts of *S. sibirica* and *S. williamsii* as the strongest ROS scavengers. Both flower extracts displayed above-average activity in all assays, while the corresponding leaf extracts performed strongly in three of the four. Conversely, both *S. nigra* extracts showed unexpectedly high mean *z*-scores, indicating below-average scavenging potential.

### Biological effects in human neutrophils

The biological segment of the study employed *f*MLP-stimulated human neutrophils as a cellular model of oxidative stress and inflammation. To ensure that the extracts and reference compounds did not exert cytotoxic effects that might confound the results, neutrophil viability at the intendent highest concentration of the extracts was assessed. After 24 h incubation, percentage of the viable cells showed no statistically significant differences compared to untreated controls (Figure S4). All analytes were therefore deemed non-cytotoxic under the experimental conditions.

**Fig. 4:**
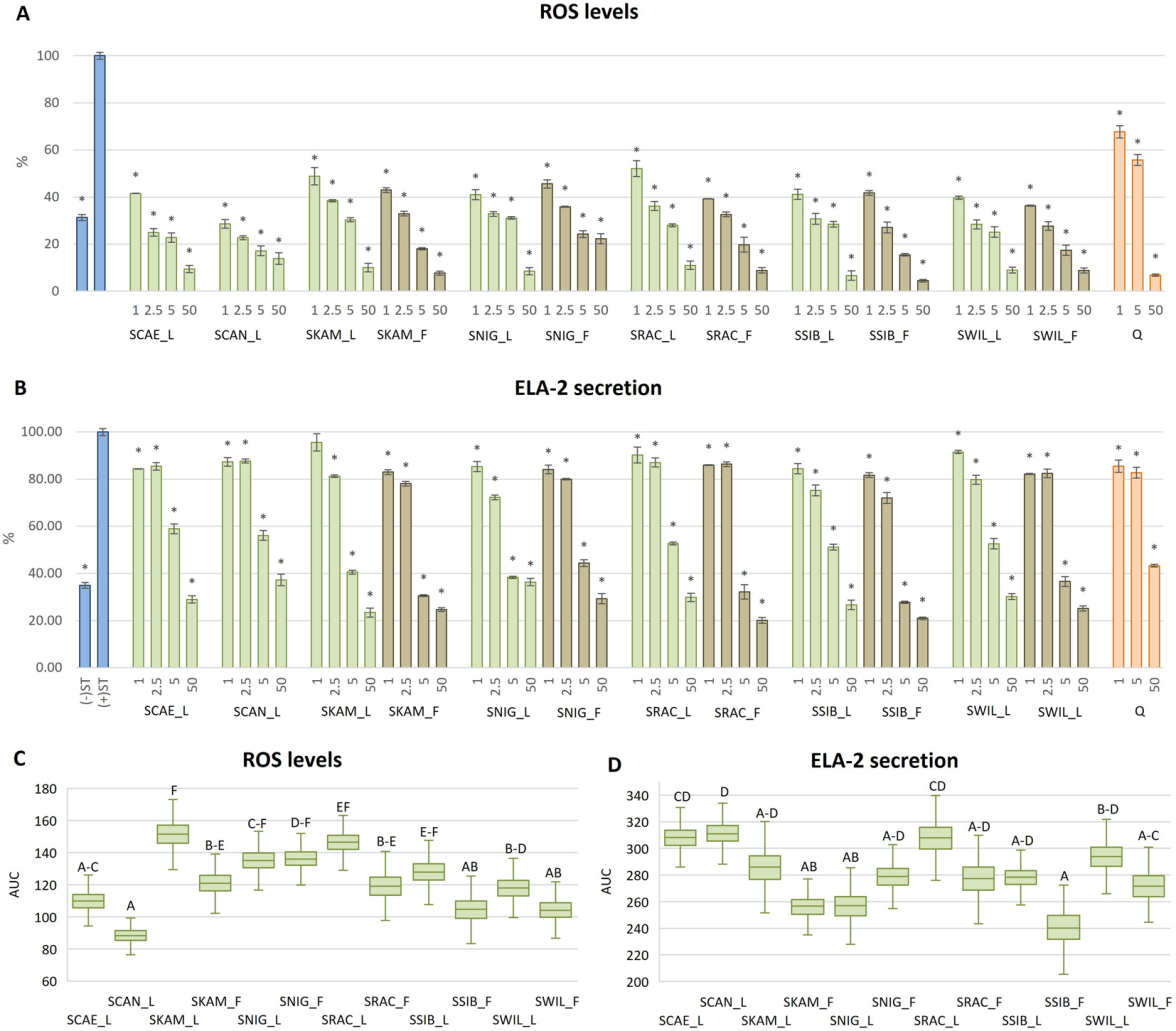
Biological activity of the investigated *Sambucus* extracts (1–50 µg/mL) in the model of human neutrophils ex vivo. (**A**) Inhibition of reactive oxygen species (ROS) release from *f*MLP-stimulated neutrophils; (**B**) inhibition of elastase (ELA-2) release from *f*MLP-cytochalasin B-stimulated neutrophils; results presented as means ± SE (*n* = 5); the statistical differences between means were assessed using Welch’s ANOVA followed by Games-Howell’s post-hoc test; *p < 0.05 compared with the stimulated control, ( +)ST; Q, quercetin (positive control); (−)ST, not stimulated cells without addition of extract/standard. Estimation of the area under the curve (AUC) across three lowest concentration (1–5 µg/mL) for (**C**) ROS release inhibition and (**D**) ELA-2 release inhibition. The boxes represent the interquartile range (IQR), with the line inside indicating the median. Whiskers extend to the minimum and maximum values within 1.5 times the IQR from the quartiles. Boxes sharing the same capital letter are not significantly different at *α* = 0.05. Refer to Table 1 for extract abbreviation. Lower AUC values indicate lower ROS/ELA-2 levels and therefore stronger inhibitory activity, higher AUC values correspond to weaker inhibition.

All extracts exhibited pronounced, concentration-dependent inhibition of ROS release (by 47.9–95.5%). Even at 1 µg/mL, inhibition ranged from 47.9% (*S. racemosa* leaf extract) to 71.3% (*S. canadensis* leaf extract), surpassing the activity of the positive control quercetin at concentrations of 1–5 µM (32.3–44.4%). At 50 µg/mL, the extracts inhibited ROS production by 77.67–95.24%, approaching or matching the effect of quercetin at 50 µM and reducing ROS levels below the baseline observed for unstimulated neutrophils (Fig. 4A).

Because the extracts relative potency varied across the tested concentrations, an integrated measure of activity was obtained by estimating the area under the curve (AUC) for activity across three lowest concentration (1–5 µg/mL). Although confidence intervals were wide and many differences were not statistically significant, clear trends were recognizable (Fig. 4C). The *S. canadensis* leaf extract showed the highest overall activity, followed by the flower extracts of *S. williamsii* and *S. sibirica* as well as *S. caerulea* leaf extract. Conversely, the weakest activity was observed for leaf extracts of *S. kamtschatica* and *S. racemosa*. Both flower and leaf extracts of *S. nigra* were ranked below the average in the assay.

The extracts also exerted a significant and dose-dependent inhibitory effect on ELA-2 secretion. At the lowest extract concentrations (1–2.5 µg/mL), ELA-2 release was inhibited by up to 28.03%, with an average activity comparable to that of quercetin (positive control) at 1–5 µM (14.54–17.26%). At 5 µg/mL, the inhibitory activity increased substantially (41.00% to 72.20%) and exceeded that of quercetin at low concentrations (1–5 µM). At 50 µg/mL, ELA-2 release was inhibited by 62.76–79.85%, comparable to or exceeding the effect of quercetin at 50 µM (Fig. 4B).The relative inhibitory potency of the extracts based on the estimated AUC values (1–5 µg/mL) is shown in Fig. 4D. The most potent inhibitor of ELA-2 secretion was the *S. sibirica* flower extract, followed by the *S. kamtschatica* flower extract and *S. nigra* leaf extract. The lowest activity was observed for the leaf extracts of *S. caerulea*, *S. canadensis* and *S. racemosa*.

## Discussion

### Chemical composition

The phenolic composition of *S. nigra* flowers has been extensively documented^36–38^, with more recent studies also addressing its leaves^12,13^. By contrast, information on other *Sambucus* species remains scarce, and comparative analyses are limited. Previous studies either focused only on total phenolic content assessed by spectrophotometric methods^24^ or included only a small number of species in LC-MS based analyses^18^. In the present work, we analyzed the extracts from leaves and flowers of seven *Sambucus* species using extensive LC-MS profiling to contribute to understanding of the phytochemical and pharmacological potential of this genus.

Direct quantitative comparison with previously published data is difficult, as reported concentrations depend strongly on extraction procedures, purification steps, analytical techniques, and the basis used for expressing results (e.g., dry plant material vs. dry extract). For *S. nigra*, the species for which the most extensive literature data are available, the concentrations observed in this study tend to be higher than the most commonly reported ranges^1,12^ and more closely resemble values obtained for partially purified extracts^39^. This difference may be related to the chloroform pre-extraction step applied in the present workflow, which likely removed lipophilic ballast components and thereby resulted in relative enrichment of phenolic constituents in the final extracts. For this reason, explicit quantitative comparisons between studies would be avoided, and the discussion will instead focus on relative trends observed among species within the scope of a single study.

Overall, the extracts investigated in this study exhibited broadly similar polyphenolic profiles dominated by quinic acid pseudodepsides and flavonol glycosides. Moreover, the extracts contained none or only negligible amounts of flavan-3-ols, both low-molecular-weight ones and condensed proanthocyanidins. These findings are in agreement with published data on diverse *Sambucus* species^1,16,18,19^, suggesting a tendency toward comparable phenolic composition within the genus.

On the other hand, previously observed for fruits^22–24^, *Sambucus* species can exhibit substantial quantitative variability. Our study confirms this observation, with the richest extracts containing nearly fourfold higher levels of phenolics than the poorest. Considering that all species were grown in close geographical proximity and sampled simultaneously, these discrepancies are likely attributable to inherent differences between species under the applied experimental conditions; however, the data collected thus far do not allow for broad generalization. For instance, in line with our findings, studies on *Sambucus* species grown in Slovenia demonstrated that flowers and fruits of *S. caerulea*, were a considerably poorer source of phenolics than those of *S. nigra*^22,24^. Conversely, *S. racemosa* flowers, relatively polyphenol-poor in our study, were previously reported to accumulate comparable levels of phenolics to *S. nigra* flowers grown at the same site^18^.

Consistent with previous observations for *S. nigra*^1,6,18^, the extracts from flowers of the investigated *Sambucus* species were typically richer in phenolics than corresponding leaf extracts. Similar trends were reported, e.g., in previous HPLC analyses of *S. ebulus*^18,39^. The exception to this pattern was again observed for *S. racemosa* samples from Slovenia, in which flower phenolic content was comparable to that of the leaves^18^. Despite being poorer sources of phenolics than corresponding flowers, leaf extracts of *S. williamsii* and *S. sibirica* still ranked among the most polyphenol-rich samples in our study. Previous studies also indicated that leaves of these species accumulate higher levels of phenolics than branches and fruits^16^. Considering their extended availability throughout the growing season and higher biomass yields relative to flowers, these leaves may represent useful sources of phenolics for further phytochemical investigation and potential applications.

Moreover, in the present study, notable qualitative differences were observed within the flavonoid fraction. Extracts of *S. nigra*, *S. williamsii*, *S. kamtschatica*, and *S. canadensis* were characterized by quercetin 3-*O*-rutinoside dominance, a feature consistently reported for *S. nigra* across multiple varieties^37,38^. In contrast, PCA and HCA showed that *S. sibirica*, *S. caerulea*, and *S. racemosa* preferentially accumulated flavonol glucosides rather than the rutinosides. This observation may have potential taxonomic relevance in the context of the still-problematic classification of the *Sambucus* genus^40^. Moreover, it may impact biological activity, as flavonoid glucosides generally exhibit higher bioavailability than the rutinosides due to more efficient intestinal hydrolysis^41,42^. Glucosides-rich species may, offer some advantages in the context of oral administration, although this would require further confirmation. To date, analogous quercetin 3-*O*-β-d-glucopyranoside dominance was observed for fruits of *S. caerulea*^43^ and flowers of *S. racemosa*^18^. In *S. racemosa* leaves, quercetin 3-*O*-rutinoside levels exceeded that of 3-*O*-β-d-glucopyranoside, but the analysis did not account for acetylated hexosides, which may explain the apparent discrepancy with our findings^18^. Conversely, *S. sibirica* leaves investigated previously showed a clear quercetin 3-*O*-rutinoside dominance^16^, suggesting that phytochemical patters in *Sambucus* species may be highly context-dependent, and warranting further investigation.

It should be noted that all results discussed above are expressed per gram of dry extract. As the extraction yields obtained for different species and plant organs ranged from 18.32 to 34.53%, the corresponding levels of analyzed metabolites in the plant material itself would be affected accordingly and this fact should be taken into account when extrapolating the data. At the same time, the observed differences in phenolic composition were generally more pronounced than the differences in extraction yield, suggesting that the relative ranking of samples would not be substantially altered when expressed on a plant material basis. However, as extraction yields were determined from a single extraction, these values should be considered approximate. Further studies using optimized and replicated extraction procedures would be required to fully assess the suitability of particular species and plant organ for large-scale extraction.

### Safety considerations

Besides phenolic compounds, vegetative organs of *Sambucus* may also contain secondary metabolites with potential toxicological relevance, such as cyanogenic glycosides. Considering this, the presence of sambunigrin was evaluated qualitatively during LC–MS profiling using an authentic reference standard. No signal corresponding to sambunigrin was observed under the applied analytical conditions, which is consistent with the known instability of cyanogenic glycosides during drying and heated extraction^44^. This observation should be interpreted with caution, as no dedicated detection limit was established, and targeted quantification of cyanogenic glycosides would be required to fully assess safety aspects, particularly since different *Sambucus* species may vary in their tendency to accumulate these compounds^18,45^.

### Biological activity

*Sambucus nigra* flowers are traditionally used as a diaphoretic and immune-supportive remedies in management of fever and upper respiratory tract infections^1,46^. More recently, its leaves have attracted attention for topical applications in skin conditions^12,13^. The molecular mechanisms underlying their efficacy include antioxidant, anti-inflammatory, and immunostimulatory effects. Given the abundance of polyphenols in *Sambucus* species, the involvement of antioxidant mechanisms is likely to contribute to their biological action, including anti-inflammatory effects and protection of immune cells.

Immune cells are among the main sources of ROS in the human body, particularly during the oxidative burst of activated neutrophils recruited to the site of the infection^47^. During this process, O_2_^−^ is generated first and then enzymatically transformed to H_2_O_2_ and HClO, among others. These ROS are crucial for pathogen elimination; however, failure of endogenous antioxidant defenses can lead to their uncontrolled generation and oxidative damage to essentials molecules^48^. Immune cells themselves are particularly vulnerable due to high content of polyunsaturated fatty acids in their membranes^47^. Therefore, chronic oxidative stress may impair their function and lead to immunosuppression and heightened susceptibility to infections^49^. Exogenous antioxidants from plant sources may therefore support host immunity by mitigating ROS-induced cellular damage^47^.

The antioxidant potential of *Sambucus* materials has been previously demonstrated in simple chemical assays for *S. nigra* flowers^6,24^ and leaves of *S. sibirica*^16^ and *S. williamsii*^50^. Moreover, *S. nigra* flower extract has been shown to reduce NO production in LPS-stimulated macrophages^5^, while *S. nigra* leaf extract has been reported to scavenge ROS, including H_2_O_2_, O_2_^−^ and NO, and to inhibit ROS release from *f*MLP-stimulated neutrophils^12^. Taking into account previous results, our comparative study evaluated both direct ROS-scavenging activity and the impact on prooxidant functions of human neutrophils, manifested in the oxidative burst.

The extracts tested here demonstrated concentration-dependent scavenging activity against HClO, OH˙, H_2_O_2_, and O_2_^−^, although their potency varied substantially among species and plant organs. The *S. nigra* leaf extract, previously reported as active against H_2_O_2_ and O_2_^−^^12^, was in our analyses outperformed by several other *Sambucus* extracts, indicating that species other than *S. nigra* may also represent promising sources of antioxidant activity. Particularly noteworthy was the high efficacy against HClO and OH˙, two of the most reactive oxidants generated in biological systems. Both species are derived from H_2_O_2_, with HClO formed by myeloperoxidase during the oxidative burst of neutrophils and OH˙ generated through the Fenton reaction between H_2_O_2_ and ferrous ions^51^. The main target of HClO are sulfur-containing peptides and proteins, whereas OH˙ preferentially attacks unsaturated fatty acids. Unlike H_2_O_2_ and O_2_^−^, which can be efficiently neutralized by enzymatic systems, no specific enzymes exist for elimination of HClO or OH˙. Their detoxification therefore depends heavily on non-enzymatic antioxidants such as AA^52^. The finding that some of the studied extracts displayed the activity comparable to or exceeding that of AA indicates their potential as exogenous antioxidants.

The in vitro assay with *f*MLP-stimulated neutrophils further supports their antioxidant efficiency. Remarkably, the extracts exhibited substantial effects even at the lowest tested concentration of 1 µg/mL, corresponding to approximately 0.2–1 µM of quinic acid pseudodepsides and 0.06–0.6 µM of flavonoids depending on the extract composition. These levels are in a similar range to plasma concentrations of caffeoylquinic acids and quercetin equivalents reported in vivo following oral ingestion of plant materials rich in these compounds^53,54^. In this context, *Sambucus* extracts may contribute to supporting immune function by limiting ROS-induced tissue damage and promoting balanced neutrophil activity.

Flavonoids and phenolic acid derivatives are also known for strengthening the capillary walls and reducing their permeability, which is beneficial for the venous system, but may also indirectly support host defense. One of the mechanisms underlying these effects is the inhibition of pro-inflammatory proteolytic enzymes responsible for degradation of the intercellular matrix^55,56^. Among the key targets is ELA-2, a serine protease primarily expressed in neutrophils, capable of degrading elastin, collagen, and fibronectin. ELA-2 is released at sites of inflammation and contributes to antimicrobial defense; however, excessive secretion promotes inflammation and infection progression by degrading the intercellular matrix and increasing capillary permeability. Additionally, it induces airway remodeling, enhances mucus secretion, and disrupts epithelial repair^57^.

In the present study, the *Sambucus* extracts inhibited ELA-2 release in a dose-dependent manner. Considering the concentrations required to achieve this effect, their activity can be regarded as moderate, however it may represent a basis for further studies addressing its physiological relevance. Only minor differences in potency were observed among the extracts, with phenolic-rich flower extracts demonstrating a slight advantage over their respective leaf counterparts. An exception was the *S. nigra* leaf extract, which exhibited relatively strong activity in this assay. This effect might be attributed to yet unidentified constituents specific to this plant material.

### Species most promising for further research

This comparative study aimed to identify *Sambucus* species with potential applications comparable to or exceeding those of *S. nigra*. Based on the results, the Asiatic species (*S. williamsii*, *S. sibirica*, *S. kamtschatica*) appear to be superior in terms of phenolic content and biological activity. Among them, *S. williamsii* and *S. sibirica* are particularly attractive due to their wide distribution and favorable agronomic traits.

*S. williamsii* is a shrub or small tree native to China and valued in traditional medicine. Its stems, rich in lignans and iridoids, are used to treat bone fractures, and have demonstrated anti-inflammatory and analgesic properties^17^. The seeds provide a valuable fixed oil rich in unsaturated fatty acids^26^. As shown in our study, *S. williamsii* flowers and leaves also represent the best alternative among the tested species to *S. nigra* plant materials in terms of composition, with similar flavonoid profile as well as high phenolic acid and quercetin 3-*O*-rutinoside content. Their antioxidant activity and elastase-inhibiting properties were among the highest compared with other *Sambucus* species investigated.

*S. sibirica* is a deciduous shrub native to Central Asia and Siberia It is used in traditional Kazakh medicine, where branches and leaves serve primarily as anti-inflammatory agents in the treatment of fractures, other injuries, rheumatoid arthritis and nephritis^16^. Our findings indicate that the flowers and leaves of *S. sibirica* contain high levels of phenolics, dominated by 5-*O*-caffeoylquinic acid and quercetin glycosides. Unlike *S. nigra* and *S. williamsii*, quercetin 3-*O*-β-d-glucopyranoside was the prevailing constituent rather than quercetin 3-*O*-rutinoside. This may offer an advantage in terms of bioavailability of *S. sibirica* phenolics; however, further studies are needed to confirm whether this pattern is consistent across the species. Moreover, similar to *S. williamsii*, *S. sibirica* ranked highly in most of the bioactivity assays.

### Limitations of the study

The present work was designed as a composite-sample comparative phytochemical and bioactivity screening of selected *Sambucus* species. As plant material was pooled prior to extraction, the results do not capture within-species variability nor can be interpreted as population-level study. Cultivation under the same environmental conditions helped minimize many potential sources of variability; however, some of the investigated species originate from different geographical regions and may not have expressed their full phytochemical potential outside their natural habitats. Moreover, the available literature indicates that phytochemical variability within the genus *Sambucus* may be substantial; therefore, the present results should be regarded as a comparative snapshot obtained under controlled analytical conditions rather than as definitive quantitative benchmarks. Further studies including material collected from multiple locations, seasons, and plant populations would be necessary to assess the stability and generality of the observed phytochemical patterns.

Another limitation concerns the extraction procedure applied in this study. The workflow was selected to obtain representative extracts suitable for phytochemical profiling and biological testing. However, the wider practical use of these species would require extractions procedures that align with the constraints of food and pharmaceutical manufacturing. Future studies should therefore explore extraction strategies using industry-compatible solvents and scalable technologies to evaluate the technological feasibility of obtaining comparable extracts.

## Conclusion

This study provides the first broad comparative evaluation of flower and leaf extracts from seven *Sambucus* species, revealing their phytochemical diversity. Although all species shared a qualitatively similar profile dominated by caffeoylquinic acids and flavonols, quantitative variations were observed between extracts of different species. These discrepancies may have practical implications for extracts composition, bioavailability and bioactivity; however, further studies are required to confirm the generality of these patterns across environmental conditions and plant populations.

The investigated extracts showed strong scavenging activity of biologically relevant ROS and significantly inhibited ROS release and ELA-2 secretion from stimulated human neutrophils. These effects may be related to the mechanisms contributing to the biological activities traditionally attributed to *Sambucus* species.

Among the investigated taxa, *S. williamsii* and *S. sibirica* were characterized by high phenolic content, favorable flavonoid composition, and strong performance in both chemical and cellular assays, in several cases exceeding *S. nigra*, a widely used European medicinal species. The combination of these advantages make these species promising for further phytochemical and pharmacological research, and potential complementary raw materials alongside *S. nigra*. Future studies should focus on more advanced in vitro and in vivo evaluation of their biological activity and optimization of extraction and processing approaches to better assess their practical applicability.

## Supplementary Information

Below is the link to the electronic supplementary material.

Supplementary Material 1

## Data Availability

Data will be made available on request.

## References

[1] Młynarczyk, K., Walkowiak-Tomczak, D. & Łysiak, G. P. Bioactive properties of *Sambucus nigra* L. as a functional ingredient for food and pharmaceutical industry. *J. Funct. Foods* **40**, 377–390 (2018). 32362939 10.1016/j.jff.2017.11.025 PMC7185606

[2] Waszkiewicz-Robak, B. & Biller, E. Właściwości prozdrowotne czarnego bzu. *Probl. Hig. Epidemiol.* **99**, 217–224 (2018).

[3] Corrado, G. et al. Cultivation, phytochemistry, health claims, and genetic diversity of *Sambucus**nigra*, a versatile plant with many beneficial properties. *Horticulturae* **9**, 488 (2023).

[4] Council of Europe. *European pharmacopoeia 11th edition* (Council of Europe, 2023).

[5] Mota, A. H. et al. Synchronous insight of in vitro and in vivo biological activities of *Sambucus nigra* L. extracts for industrial uses. *Ind. Crops Prod.* **154**, 112709 (2020).

[6] Dawidowicz, A. L., Wianowska, D. & Baraniak, B. The antioxidant properties of alcoholic extracts from *Sambucus nigra* L. (antioxidant properties of extracts). *LWT Food Sci. Technol.* **39**, 308–315 (2006).

[7] Patel, K. & Patel, D. K. The beneficial role of Rutin, a naturally occurring flavonoid in health promotion and disease prevention: A systematic review and update. In *Bioactive Food as Dietary Interventions for Arthritis and Related Inflammatory Diseases* 457–479 (Elsevier, 2019). 10.1016/B978-0-12-813820-5.00026-X.

[8] Liang, N. & Kitts, D. Role of chlorogenic acids in controlling oxidative and inflammatory stress conditions. *Nutrients* **8**, 16 (2015). 26712785 10.3390/nu8010016 PMC4728630

[9] Ding, Y. et al. Antiviral activity of chlorogenic acid against influenza A (H1N1/H3N2) virus and its inhibition of neuraminidase. *Sci. Rep.* **7**, 45723 (2017). 28393840 10.1038/srep45723 PMC5385491

[10] Scopel, M., Mentz, L. & Henriques, A. Comparative analysis of *Sambucus nigra* and *Sambucus australis* flowers: Development and validation of an HPLC method for raw material quantification and preliminary stability study. *Planta Med.* **76**, 1026–1031 (2010). 20195957 10.1055/s-0029-1240850

[11] Charlebois, D., Byers, P. L., Finn, C. E. & Thomas, A. L. Elderberry: Botany, horticulture, potential. In *Horticultural Reviews* Vol. 37 213–280 (Wiley, 2010).

[12] Skowrońska, W., Granica, S., Czerwińska, M. E., Osińska, E. & Bazylko, A. *Sambucus nigra* L. leaves inhibit TNF-α secretion by LPS-stimulated human neutrophils and strongly scavenge reactive oxygen species. *J. Ethnopharmacol.* **290**, 115116 (2022). 35182667 10.1016/j.jep.2022.115116

[13] Skowrońska, W. et al. Wound healing potential of extract from *Sambucus nigra* L. leaves and its fractions. *J. Ethnopharmacol.* **320**, 117423 (2024). 37979821 10.1016/j.jep.2023.117423

[14] Waswa, E. N. et al. Ethnobotany, phytochemistry, pharmacology, and toxicology of the genus *Sambucus* L. (Viburnaceae). *J. Ethnopharmacol.* **292**, 115102 (2022). 35288288 10.1016/j.jep.2022.115102

[15] Waswa, E. N. et al. Understanding the taxonomic complexes and species delimitation within *Sambucus* L. (Viburnaceae). *Diversity* **14**, 906 (2022).

[16] Yan, P. et al. Microscopic identification, phytochemical analysis, and study of antioxidant properties of branches, leaves, and fruits of Kazakh medicine *Sambucus sibirica*. *Molecules*10.3390/molecules29235503 (2024). 39683662 10.3390/molecules29235503 PMC11643177

[17] Xiao, H.-H., Zhang, Y., Cooper, R., Yao, X.-S. & Wong, M.-S. Phytochemicals and potential health effects of *Sambucus williamsii* Hance (Jiegumu). *Chin. Med.* **11**, 36 (2016). 27478495 10.1186/s13020-016-0106-9 PMC4965893

[18] Senica, M., Stampar, F. & Mikulic-Petkovsek, M. Harmful (cyanogenic glycoside) and beneficial (phenolic) compounds in different *Sambucus species*. *J. Berry Res.* **9**, 395–409 (2019).

[19] Lamy, S., Muhire, É. & Annabi, B. Antiproliferative efficacy of elderberries and elderflowers (*Sambucus canadensis*) on glioma and brain endothelial cells under normoxic and hypoxic conditions. *J. Funct. Foods* **40**, 164–179 (2018).

[20] Lee, J. & Finn, C. E. Anthocyanins and other polyphenolics in American elderberry (*Sambucus canadensis*) and European elderberry (*S. nigra*) cultivars. *J. Sci. Food Agric.* **87**, 2665–2675 (2007). 20836175 10.1002/jsfa.3029

[21] Jordheim, M., Giske, N. H. & Andersen, Ø. M. Anthocyanins in Caprifoliaceae. *Biochem. Syst. Ecol.* **35**, 153–159 (2007).

[22] Mikulic-Petkovsek, M. et al. Investigation of anthocyanin profile of four elderberry species and interspecific hybrids. *J. Agric. Food Chem.* **62**, 5573–5580 (2014). 24830391 10.1021/jf5011947

[23] Avula, B. et al. Chemical profiling and UHPLC-QToF analysis for the simultaneous determination of anthocyanins and flavonoids in *Sambucus* berries and authentication and detection of adulteration in elderberry dietary supplements using UHPLC-PDA-MS. *J. Food Compos. Anal.* **110**, 104584 (2022).

[24] Mikulic-Petkovsek, M., Ivancic, A., Schmitzer, V., Veberic, R. & Stampar, F. Comparison of major taste compounds and antioxidative properties of fruits and flowers of different *Sambucus* species and interspecific hybrids. *Food Chem.* **200**, 134–140 (2016). 26830570 10.1016/j.foodchem.2016.01.044

[25] POWO. Plants of the World Online. Facilitated by the Royal Botanic Gardens, Kew. https://powo.science.kew.org/ (2025).

[26] ICH. ICH Q2(R2) Guideline on Validation of Analytical Procedures. (2023).

[27] Porter, L. J., Hrstich, L. N. & Chan, B. G. The conversion of procyanidins and prodelphinidins to cyanidin and delphinidin. *Phytochemistry* **25**, 223–230 (1985).

[28] Owczarek, A. et al. Potential activity mechanisms of *Aesculus hippocastanum* bark: Antioxidant effects in chemical and biological in vitro models. *Antioxidants* **10**, 995 (2021). 34206691 10.3390/antiox10070995 PMC8300635

[29] Böyum, A. A one-stage procedure for isolation of granulocytes and lymphocytes from human blood. General sedimentation properties of white blood cells in a 1g gravity field. *Scand. J. Clin. Lab. Invest.* **Suppl. 97**, 51–76 (1968). 4179067

[30] Michel, P. et al. Salicylate and procyanidin-rich stem extracts of *Gaultheria procumbens* L. Inhibit pro-inflammatory enzymes and suppress pro-inflammatory and pro-oxidant functions of human neutrophils ex vivo. *Int J Mol Sci* **20**, 1753 (2019). 30970662 10.3390/ijms20071753 PMC6479601

[31] Magiera, A. et al. Polyphenol-enriched extracts of prunus spinosa fruits: anti-inflammatory and antioxidant effects in human immune cells ex vivo in relation to phytochemical profile. *Molecules* **27**, 1691 (2022). 35268792 10.3390/molecules27051691 PMC8912089

[32] Clifford, M. N., Johnston, K. L., Knight, S. & Kuhnert, N. Hierarchical scheme for LC-MS ^*n*^ identification of chlorogenic acids. *J. Agric. Food Chem.* **51**, 2900–2911 (2003). 12720369 10.1021/jf026187q

[33] Castillo-Fraire, C. M., Poupard, P., Guilois-Dubois, S., Salas, E. & Guyot, S. Preparative fractionation of 5′-O-caffeoylquinic acid oxidation products using centrifugal partition chromatography and their investigation by mass spectrometry. *J. Chromatogr. A* **1592**, 19–30 (2019). 30738615 10.1016/j.chroma.2019.01.071

[34] Lin, L.-Z., Sun, J., Chen, P., Monagas, M. J. & Harnly, J. M. UHPLC-PDA-ESI/HRMS ^*n*^ profiling method to identify and quantify oligomeric proanthocyanidins in plant products. *J. Agric. Food Chem.* **62**, 9387–9400 (2014). 25032782 10.1021/jf501011y PMC4181120

[35] Clifford, M. N., Knight, S. & Kuhnert, N. Discriminating between the six isomers of Dicaffeoylquinic acid by LC-MS ^*n*^. *J. Agric. Food Chem.* **53**, 3821–3832 (2005). 15884803 10.1021/jf050046h

[36] Ferreira, S. S., Silva, A. M. & Nunes, F. M. *Sambucus nigra* L. fruits and flowers: Chemical composition and related bioactivities. *Food Rev. Int.* **38**, 1–29 (2020).

[37] Christensen, L. P., Kaack, K. & Fretté, X. C. Selection of elderberry (*Sambucus nigra* L.) genotypes best suited for the preparation of elderflower extracts rich in flavonoids and phenolic acids. *Eur. Food Res. Technol.* **227**, 293–305 (2008).

[38] Vrchotová, N., Dadáková, E., Matějíček, A., Tříska, J. & Kaplan, J. Effect of variety on content of bioactive phenolic compounds in common elder (*Sambucus nigra *L.). *Nat. Prod. Res.* **31**, 700–703 (2017). 27484408 10.1080/14786419.2016.1214826

[39] Seymenska, D. et al. Comparative Study on phytochemical composition, antioxidant, and Anti-HSV-2 activities of *Sambucus nigra *L and *Sambucus ebulus Extracts*. *Appl Sci* **13**, 12593 (2023).

[40] Waswa, E. N. et al. Comparative chloroplast genome analysis of *Sambucus* L. (Viburnaceae): inference for phylogenetic relationships among the closely related *Sambucus* adnate. Wall ex DC Sambucus Javanica Blume. *Front. Plant Sci.* **14**, 1179510 (2023). 37396648 10.3389/fpls.2023.1179510 PMC10313135

[41] Murota, K. et al. α-Oligoglucosylation of a sugar moiety enhances the bioavailability of quercetin glucosides in humans. *Arch. Biochem. Biophys.* **501**, 91–97 (2010). 20638359 10.1016/j.abb.2010.06.036

[42] Makino, T. et al. Enzymatically modified isoquercitrin, ALPHA-oligoglucosyl quercetin 3-O-glucoside, is absorbed more easily than other quercetin glycosides or aglycone after oral administration in ra ts. *Biol. Pharm. Bull.* **32**, 2034–2040 (2009). 19952424 10.1248/bpb.32.2034

[43] Mikulic-Petkovsek, M., Ivancic, A., Todorovic, B., Veberic, R. & Stampar, F. Fruit phenolic composition of different elderberry species and hybrids. *J. Food Sci.* **80**, C2180–C2190 (2015). 26409176 10.1111/1750-3841.13008

[44] Bolarinwa, I. F. et al. A review of cyanogenic glycosides in edible plants. *Toxicol New Aspects Sci Conundrum*10.5772/64886 (2016).

[45] Buhrmester, R. A., Ebinger, J. E. & Seigler, D. S. Sambunigrin and cyanogenic variability in populations of *Sambucus canadensis* L. (Caprifoliaceae). *Biochem. Syst. Ecol.* **28**, 689–695 (2000). 10854744 10.1016/s0305-1978(99)00105-2

[46] European Medicines Agency. Assessment Report on Sambucus Nigra L., Flos. www.ema.europa.eu/contact (2018).

[47] Puertollano, M. A., Puertollano, E., Alvarez de Cienfuegos, G. & de Pablo, M. A. Dietary antioxidants: Immunity and host defense. *Curr. Top. Med. Chem.* **11**, 1752–1766 (2011). 21506934 10.2174/156802611796235107

[48] Andrés, C. M. C., Pérez de la Lastra, J. M., Juan, C. A., Plou, F. J. & Pérez-Lebeña, E. The role of reactive species on innate immunity. *Vaccines* **10**, 1735 (2022). 36298601 10.3390/vaccines10101735 PMC9609844

[49] Morris, G., Gevezova, M., Sarafian, V. & Maes, M. Redox regulation of the immune response. *Cell. Mol. Immunol.* **19**, 1079–1101 (2022). 36056148 10.1038/s41423-022-00902-0 PMC9508259

[50] Seo, K. & Yun, K. W. In vitro antimicrobial and antioxidant activities of *Sambucus williamsii* and *Sambucus pendula*. *Int J Secondary Metabolite* **11**, 191–199 (2024).

[51] Winterbourn, C. C., Kettle, A. J. & Hampton, M. B. Reactive oxygen species and neutrophil function. *Annu. Rev. Biochem.* **85**, 765–792 (2016). 27050287 10.1146/annurev-biochem-060815-014442

[52] Andrés, C. M. C., Pérez de la Lastra, J. M., Juan, C. A., Plou, F. J. & Pérez-Lebeña, E. Hypochlorous acid chemistry in mammalian cells—Influence on infection and role in various pathologies. *Int. J. Mol. Sci.* **23**, 10735 (2022). 36142645 10.3390/ijms231810735 PMC9504810

[53] Manach, C., Williamson, G., Morand, C., Scalbert, A. & Rémésy, C. Bioavailability and bioefficacy of polyphenols in humans. I. Review of 97 bioavailability studies. *Am. J. Clin. Nutr.* **81**, 230S-242S (2005). 15640486 10.1093/ajcn/81.1.230S

[54] Stalmach, A., Williamson, G. & Crozier, A. Impact of dose on the bioavailability of coffee chlorogenic acids in humans. *Food Funct.* **5**, 1727–1737 (2014). 24947504 10.1039/c4fo00316k

[55] Jakimiuk, K., Gesek, J., Atanasov, A. G. & Tomczyk, M. Flavonoids as inhibitors of human neutrophil elastase. *J. Enzyme Inhib. Med. Chem.* **36**, 1016–1028 (2021). 33980119 10.1080/14756366.2021.1927006 PMC8128182

[56] Mohamed, E. M. et al. Bioassay-guided isolation, metabolic profiling, and docking studies of hyaluronidase inhibitors from *Ravenala madagascariensis*. *Molecules* **25**(7), 1714 (2020). 32276509 10.3390/molecules25071714 PMC7180949

[57] Zeng, W., Song, Y., Wang, R., He, R. & Wang, T. Neutrophil elastase: From mechanisms to therapeutic potential. *J. Pharm. Anal.* **13**, 355–366 (2023). 37181292 10.1016/j.jpha.2022.12.003 PMC10173178

