# Supplementary material for: Multivariate comparative assessment of extracts from seven Sambucus species reveals phytochemical diversity and biological potential
Source: Sci Rep. 2026 Apr 28;16:19649. doi: 10.1038/s41598-026-50152-7 (PMC13315254; doi:10.1038/s41598-026-50152-7)
Supplement: Supplementary file 1 — Supplementary Material 1 [file 41598_2026_50152_MOESM1_ESM.pdf]

Multivariate comparative assessment of extracts from seven Sambucus species reveals phytochemical diversity and biological potential

Aleksandra Owczarek-Januszkiewicz[a,*], Anna Magiera[a], Sebastian Granica[b], Gabriela Cieślak[a], Magdalena Życka[a], , Izabela Rychlińska[a], Monika Anna Olszewska[a]

[a] Department of Pharmacognosy, Faculty of Pharmacy, Medical University of Lodz, Muszyńskiego 1, 90-151 Lodz, Poland

[b] Department of Pharmaceutical Biology, Faculty of Pharmacy, Warsaw Medical University, Banacha 1, 02-097 Warsaw, Poland

SUPPLEMENTARY MATERIALS

Table of content

1. Validation of the HLPC-PDA quantitative procedure 2

1.1. Materials and Methods 2

Table S1 Reference substances used for quantitative studies. 2

1.2. Statistical Analysis 2

1.3. Results 3

Table S2 Linearity and sensitivity data 3

Table S3 Precision and accuracy data 3

Figure S1. Representative UV-Vis (280 nm) chromatograms of the investigated extracts 4

Figure S1 cont. 5

Figure S2. Content of condensed proanthocyanidins in investigated extracts 6

Figure S3. Scavenging effects of the investigated *Sambucus* extracts 6

Figure S4. Effect of investigated *Sambucus* extracts on viability of human immune cells 7

References 7

1. Validation of the HLPC-PDA quantitative procedure

1.1. Materials and Methods

The validation was performed for seven fully identified constituents of *Sambuci flos*, with the authentic standard available, obtained either from commercial source or from previous isolation studies in the Department of Pharmacognosy, Medical University of Lodz (Table S1).

Table S1
Reference substances used for quantitative studies.

Abbreviation	Reference substance	Purity and source
5-CQA	5-caffeoylquinic acid (chlorogenic acid)	>98% (A)
QRT	quercetin 3-*O*-(6''-*O*-α-L-rhamnopyranosyl)-β-D-glucopyranoside (rutin)	>98% (A)
QG	quercetin 3-*O*-β-D-glucopyranoside (isoquercitrin)	>97% (A)
KRT	kaempferol 3-*O*-(6''-*O*-α-L-rhamnopyranosyl)-β-D-glucopyranoside	>98% (B)
KG	kaempferol 3-*O*-β-D-glucopyranoside (astragalin)	>98% (B)
IRT	isorhamnetin 3-*O*-(6''-*O*-α-L-rhamnopyranosyl)-β-D-glucopyranoside	>98% (B)
IG	isorhamnetin 3-*O*-β-D-glucopyranoside	>98% (B)

A, compounds purchased from Sigma Aldrich (Seelze, Germany/St. Louis, MO, USA) or Phytolab (Vestenbergsgreuth, Germany); B, compounds isolated in the Department of Pharmacognosy, Medical University of Lodz;

To test linearity, the stock solution of the mixed standards was prepared in methanol-water (7:3, *v/v*) and serially diluted with the same solvent to six concentration levels (2%, 10%, 25%, 50%, 75%, and 100% of the stock concentration). Each replicate solution was injected into the HPLC system in triplicate. The statistical significance of the regression equations was evaluated using *F*- and *t*-tests at a 99% confidence level.

The LOD and LOQ values were determined by further serial dilution of the standard solutions with methanol-water (7:3, *v/v*). The lowest concentrations with the signal-to-noise ratio (S/N) above 3 were accepted as LODs, while the levels with S/N above 10 were accepted as LOQs if the RSD values for peak area were not higher than 15%.

The repeatability (intra-day variability) and the intermediate precision (inter-day variability) were tested for retention times and peak areas using the standard solutions at 10% and 100% of the stock concentration and selected extract sample containing the given analyte at measurable levels. The repeatability was determined by triplicate analysis of each sample within 24 h, while the intermediate precision was evaluated on three non-consecutive days within two weeks.

The accuracy was tested by the standard addition/recovery procedure in the selected extract samples containing the given analyte at measurable levels. Three different levels of each standard, within the analytical range were investigated. The samples were prepared in triplicate by spiking the sample with the standard solution. The replicate samples were analysed in triplicate. The accuracy was calculated as the mean recovery of the analytes from the spiked versus the non-spiked extracts.

1.2. Statistical Analysis

The results were expressed as means ± standard deviation (SD) for replicate determinations. The statistical analyses (calculation of SD, linearity studies) were performed using the Statistica12Pl software for Windows (StatSoft Inc., Krakow, Poland), with *p* values less than 0.05 being regarded as significant.

1.3. Results

The analytical method was validated by determining the selectivity, linearity, precision, and accuracy according to the guidance of the International Council for Harmonisation (ICH, 2023).

The selectivity of the method, as well as the peak purity, were analyzed by comparison of the retention times and UV-vis spectra with reference compounds using an automatic match system. Comparisons of the spectra's upslopes, apexes, and downslopes, as well as the peak spectral data at different wavelengths, confirmed that all analyte peaks of the real samples eluted as pure bands.

The linearity of the method for the six standards was confirmed in the whole range of concentrations used with $r > 0.9995$ (Table S2). The statistical significance of the obtained regression equations was confirmed in the F-test ($p < 0.05$). Good sensitivity of the method was demonstrated by low LODs and LOQs values (Table S2).

In the precision test, the RSD values (Table S3) measured for peak area of each analyte for repeatability and intermediate precision did not exceed the predicted critical values (PRSD, 1.3-2.7%), calculated according to the requirements of AOAC International (AOAC International, 2016), which indicated that the developed method is adequately precise. In accuracy studies, the recoveries were between 96.7% and 100.8% (Table S3) and within the limits of acceptance for botanical samples (92-105%) (AOAC International, 2013).

Table S2
Linearity and sensitivity data for the HPLC-PDA method used in the quantitative studies.

Analyte[a]	t_R ± SD (min)	λ (nm)	Linearity			Sensitivity	
			Linear regression	r	Linear range (μg/mL)	LOD (μg/mL)	LOQ (μg/mL)
5-CQA	9.90 ± 0.05	325	$y = 13120.49x$	0.9997	1.64-164.1	0.10	0.30
QRT	20.13 ± 0.05	350	$y = 6864.257x$	0.9997	1.68-168.1	0.56	1.68
QG	20.92 ± 0.05	350	$y = 9158.527x$	0.9997	1.88-94.1	0.43	1.31
KRT	22.77 ± 0.13	350	$y = 5380.917x$	0.9997	2.16-109.0	0.72	2.16
KG	23.72 ± 0.04	350	$y = 9024.665x$	0.9996	1.36-68.3	0.45	1.36
IRT	23.49 ± 0.04	350	$y = 6885.812x$	0.9998	1.53-76.6	0.51	1.53
IG	24.34 ± 0.09	350	$y = 8373.653x$	0.9998	1.32-66.4	0.44	1.32

Table S3
Precision and accuracy data for the HPLC-PDA method used in the quantitative studies.

Analyte	Precision (RSD, %)				Accuracy
	Repeatability		Intermediate Precision		Mean recovery ± SD (%)
	t_R	Peak area	t_R	Peak area	
5-CQA	0.13	0.41	0.65	1.42	100.8 ± 1.3
QRT	0.04	0.29	0.36	1.67	96.7 ± 2.1
QG	0.04	0.23	0.34	2.14	98.6 ± 1.9
KRT	0.04	1.20	0.29	2.57	97.4 ± 0.9
KG	0.04	0.69	0.29	2.03	99.2 ± 1.3
IRT	0.04	0.51	0.47	1.97	97.1 ± .1.4
IG	0.04	0.75	0.27	2.47	98.1 ± 1.5

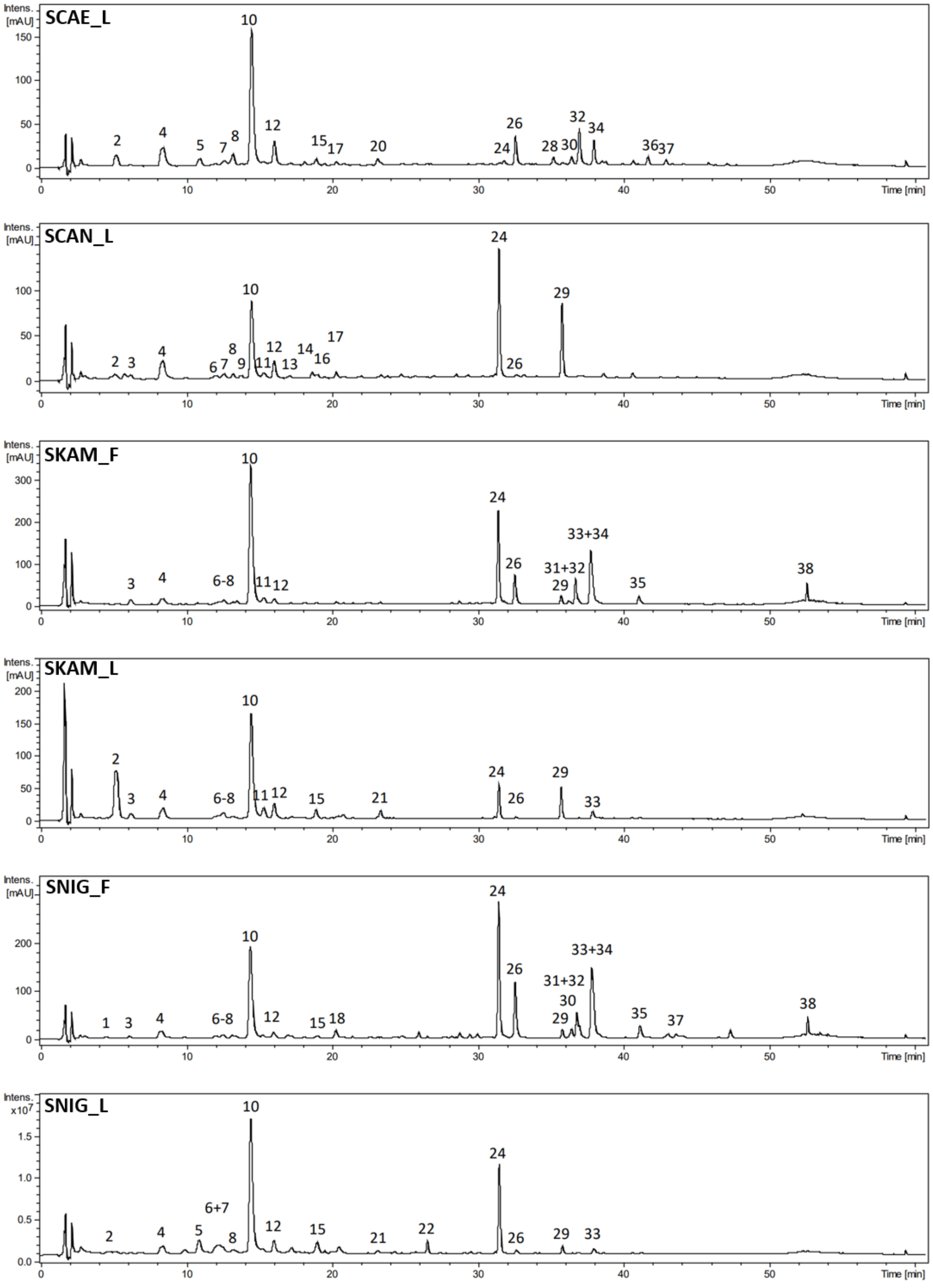

Figure S1. Representative UV-Vis (280 nm) chromatograms of the investigated extracts. Refer to Table 1 for extract abbreviations.

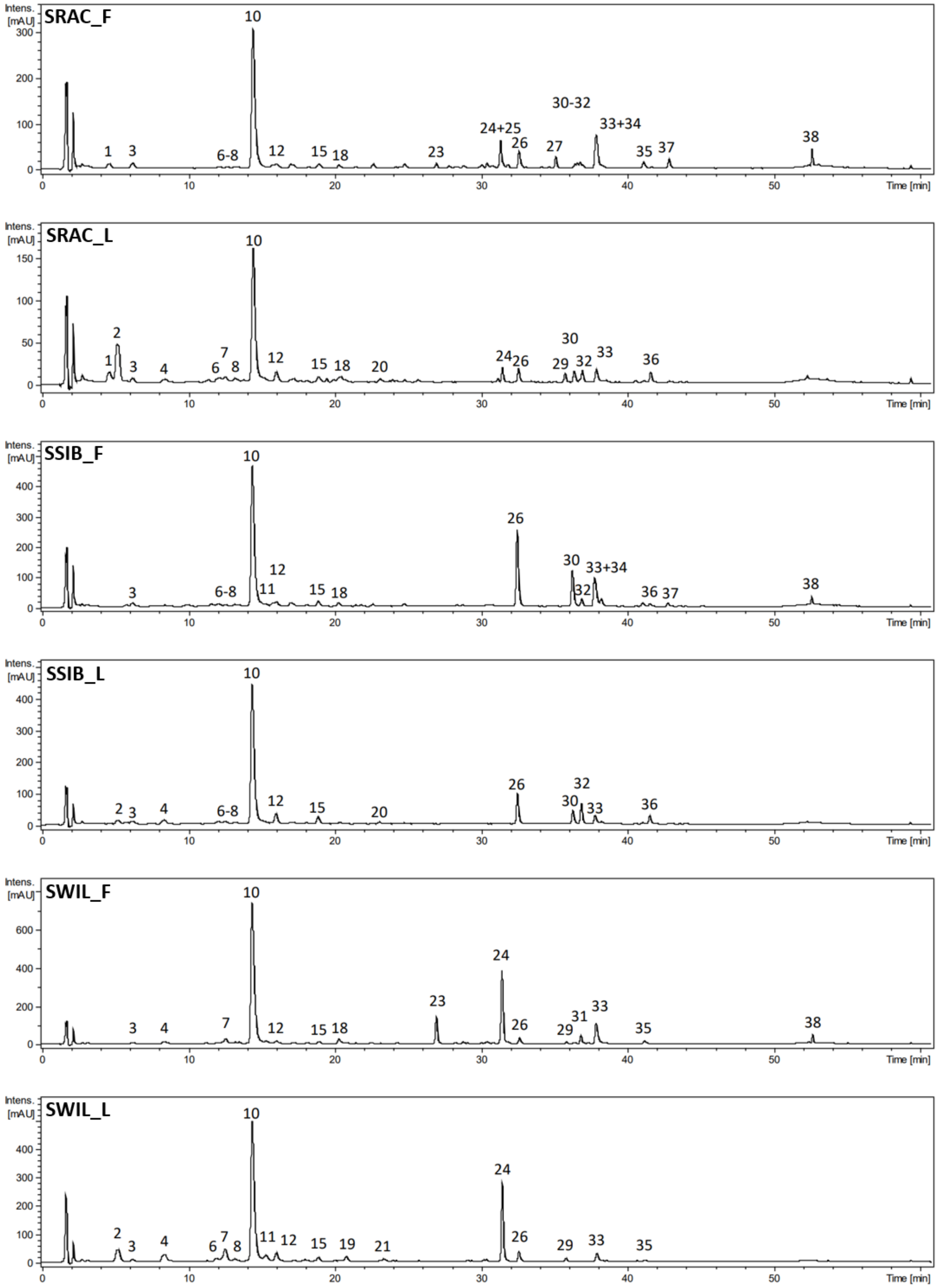

Figure S1 cont. Representative UV-Vis (280 nm) chromatograms of the investigated extracts. Refer to Table 1 for extract abbreviations

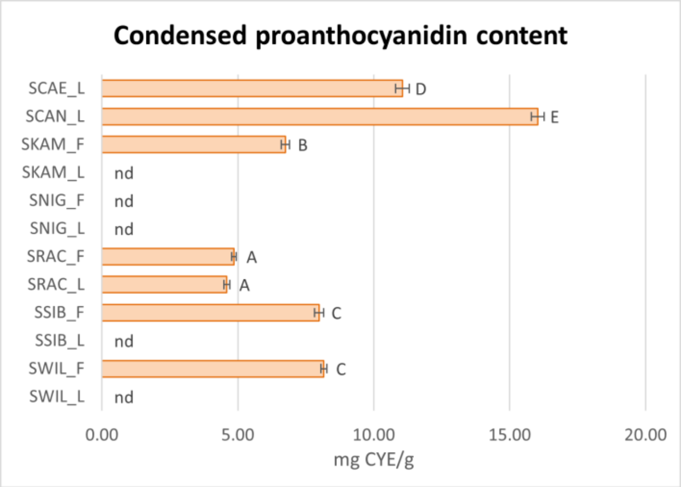

Figure S2. Content of condensed proanthocyanidins in investigated extracts quantified spectrophotometrically and expressed in mg cyanidin equivalents/g of dry extract (mg CYE/g DW). Results presented as means ± SE (n = 3). The statistical differences between means were assessed using Welch's ANOVA followed by Games-Howell's post-hoc test. Means sharing the same capital letter are not significantly different at α = 0.05. Refer to Table 1 for extract abbreviations; nd, not detected.

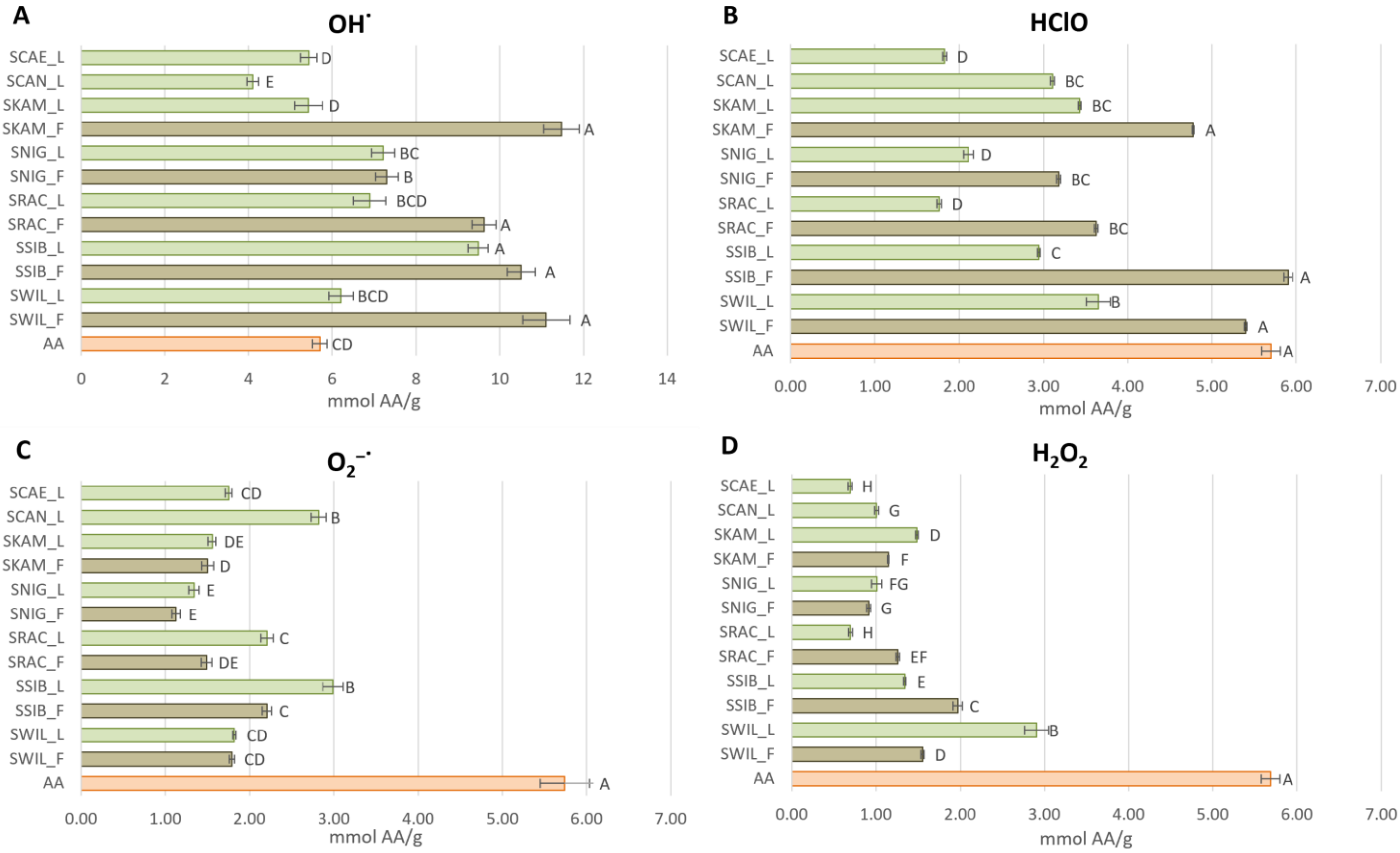

Figure S3. Scavenging effects of the investigated *Sambucus* extracts and ascorbic acid (AA, positive control) towards (A) hydroxyl radical ($OH^{\bullet}$); (B) hypochlorous acid (HClO); (C) superoxide anion radical ($O_2^{\bullet -}$) and (D) hydrogen peroxide (H_2O_2). Results expressed in millimolar equivalents of AA per g of dry extracts (mmol AA/g) and presented as means ± SE (n = 5). The statistical differences between means were assessed using Welch's ANOVA followed by Games-Howell's post-hoc test. Means sharing the same capital letter for a given radical are not significantly different at α = 0.05. Refer to Table 1 for extracts abbreviations.

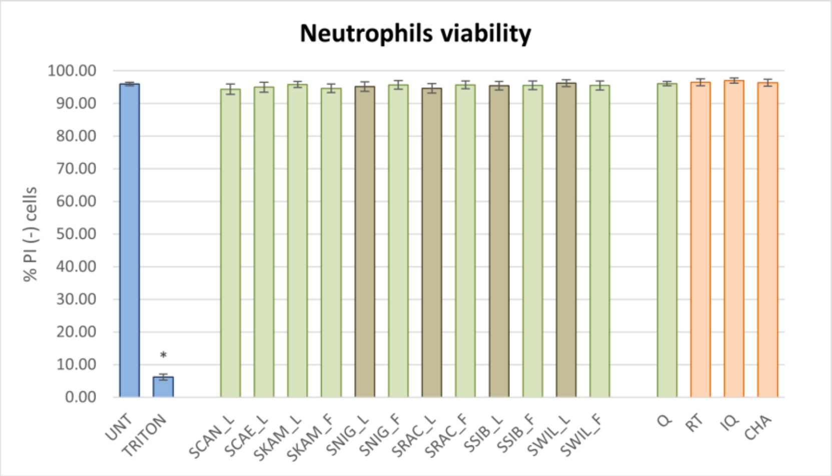

Figure S4. Effect of investigated *Sambucus* extracts (50 µg/mL) and standards (50 µM) on viability (membrane integrity) of human immune cells expressed as a percentage of PI(−) cells (propidium iodide-negative) after 24 h incubation. Results presented as means ± SE ($n = 5$). The statistical differences between means were assessed using Welch's ANOVA followed by Games-Howell's post-hoc test; * $p < 0.001$ compared with the untreated control (UNT). Refer to Table 1 for extracts abbreviations; standards: Q, quercetin; RT, rutin; IQ, isoquercitrin; CHA, chlorogenic acid; positive control: Triton X-100 solution.

References

AOAC International, 2016. Appendix F: Guidelines for Standard Method Performance Requirements. AOAC Official Methods of Analysis.

AOAC International, 2013. Appendix K: Guidelines for Dietary Supplements and Botanicals. AOAC Official Methods of Analysis.

ICH, 2023. ICH Q2(R2) Guideline on validation of analytical procedures. Amsterdam.
